# Oncogenic *RAS* Enables DNA Damage- and p53-Dependent Differentiation of Acute Myeloid Leukemia Cells in Response to Chemotherapy

**DOI:** 10.1371/journal.pone.0007768

**Published:** 2009-11-05

**Authors:** Mona Meyer, Daniela Rübsamen, Robert Slany, Thomas Illmer, Kathleen Stabla, Petra Roth, Thorsten Stiewe, Martin Eilers, Andreas Neubauer

**Affiliations:** 1 Klinik für Hämatologie, Onkologie und Immunologie, Philipps Universität Marburg, Marburg, Germany; 2 Institut für Molekularbiologie und Tumorforschung, Philipps Universität Marburg, Marburg, Germany; 3 Institut für Genetik, Friedrich Alexander Universität Erlangen, Erlangen, Germany; 4 Technische Universität Dresden, Medizinische Klinik I, Dresden, Germany; 5 Biocenter, Julius-Maximilians-Universität Würzburg, Würzburg, Germany; National Cancer Institute, United States of America

## Abstract

Acute myeloid leukemia (AML) is a clonal disease originating from myeloid progenitor cells with a heterogeneous genetic background. High-dose cytarabine is used as the standard consolidation chemotherapy. Oncogenic *RAS* mutations are frequently observed in AML, and are associated with beneficial response to cytarabine. Why AML-patients with oncogenic *RAS* benefit most from high-dose cytarabine post-remission therapy is not well understood. Here we used bone marrow cells expressing a conditional *MLL-ENL-ER* oncogene to investigate the interaction of oncogenic *RAS* and chemotherapeutic agents. We show that oncogenic *RAS* synergizes with cytotoxic agents such as cytarabine in activation of DNA damage checkpoints, resulting in a p53-dependent genetic program that reduces clonogenicity and increases myeloid differentiation. Our data can explain the beneficial effects observed for AML patients with oncogenic *RAS* treated with higher dosages of cytarabine and suggest that induction of p53-dependent differentiation, e.g. by interfering with Mdm2-mediated degradation, may be a rational approach to increase cure rate in response to chemotherapy. The data also support the notion that the therapeutic success of cytotoxic drugs may depend on their ability to promote the differentiation of tumor-initiating cells.

## Introduction

Acute myeloid leukemia (AML) is a clonal disease with a heterogeneous genetic background. Besides age, cytogenetic alterations and molecular lesions such as mutations in the FLT-3 or nucleophosmin genes play a pivotal role in predicting treatment response ([1]; reviewed in [2], [3]). AML is treated with induction and post-remission chemotherapy, frequently including high-dose cytarabine [4]; reviewed in: [5]. Mutations in *NRAS* and *KRAS* protooncogenes (resulting in “oncogenic” *RAS*) occur in approximately 20% of AML cases (reviewed in: [6]).

It has been suggested that leukemic transformation depends on the occurrence of two genetic lesions in a susceptible progenitor cell. Class I mutations that affect genes encoding receptor tyrosine kinases (Flt-3 or Kit) or *RAS* are thought to induce myeloid proliferation. Class II lesions affect transcription factors such as nucleophosmin, C/EBPα, AML-ETO, MLL-ENL, PML-RARα and block differentiation (e.g. [7]; reviewed in: [3]). Supporting this notion, oncogenic *RAS* alone induces a myeloproliferative state in murine models [8]–[12] and in cooperation with nuclear oncogenes such as *PML-RAR*α induces acute leukemia [13].

Oncogenic *RAS* can also promote the differentiation of hematopoietic and other cells [14]–[18]. In non-hematopoietic cells such as primary fibroblasts, oncogenic *RAS* induces a permanent growth arrest termed senescence, which limits *RAS*-induced tumorigenesis *in vivo* [19]–[23]. Induction of senescence by oncogenic *RAS* involves activation of DNA damage checkpoints and is mediated by p53 [24], [25]. Therefore, in presence of functional p53 oncogenic *RAS* activates a “*fail-safe*” mechanism, which protects from *RAS*-induced malignant transformation.

A previous landmark study showed that AML patients benefit from high-dose cytarabine as post-induction therapy, which has subsequently become the standard consolidation therapy in AML [4]. Using AML samples taken from this study, we have previously shown that AML patients harboring oncogenic *RAS* show significantly less cumulative incidence of relapse upon treatment with high-dose cytarabine in the post-induction chemotherapy (best group), when compared to AML patients with oncogenic *RAS* treated with low-dose cytarabine (worst group). In contrast, dose escalation had a much weaker effect on the response to cytarabine in patients that harbour wild type *RAS* (intermediate groups [26]). These data suggested that there is a genetic interaction between the dose of cytarabine and the presence of oncogenic *RAS*. Importantly, multivariate analysis revealed that the interaction of *RAS* with cytarabine dose escalation was independent of cytogenetic status of the leukemic blasts, suggesting that oncogenic *RAS* affects the response of AML blasts to cytarabine [26]. Moreover, since the beneficial effect of high-dose cytarabine with oncogenic *RAS* is observed especially in the post-induction therapy, the findings suggested that the number of clonogenic leukemia-initiating cells was reduced as the result of an interaction between oncogenic *RAS* and cytarabine.

To better understand the interaction of *RAS* with cytarabine, we expressed oncogenic *RAS* in primary mouse bone marrow stem cells that had been immortalized by a MLL-ENL oncogene [27]. In this tissue-culture system, MLL-ENL acts as the class II mutation, whereas supplementation of the medium with growth factors presumably substitutes for a class I mutation. We find that oncogenic *RAS* in combination with DNA-damaging agents such as cytarabine decreases the clonogenic potential of these cells and induces a myeloid differentiation program in a DNA damage checkpoint- and p53-dependent manner. Our data suggest that in AML patients with oncogenic *RAS*, high-dose cytarabine therapy is effective since it promotes the differentiation of tumor-initiating cells.

## Results

### Immortalized bone marrow stem cells expressing oncogenic *RAS* do not show enhanced proliferation, apoptosis or senescence in response to cytarabine

**Figure 1:**
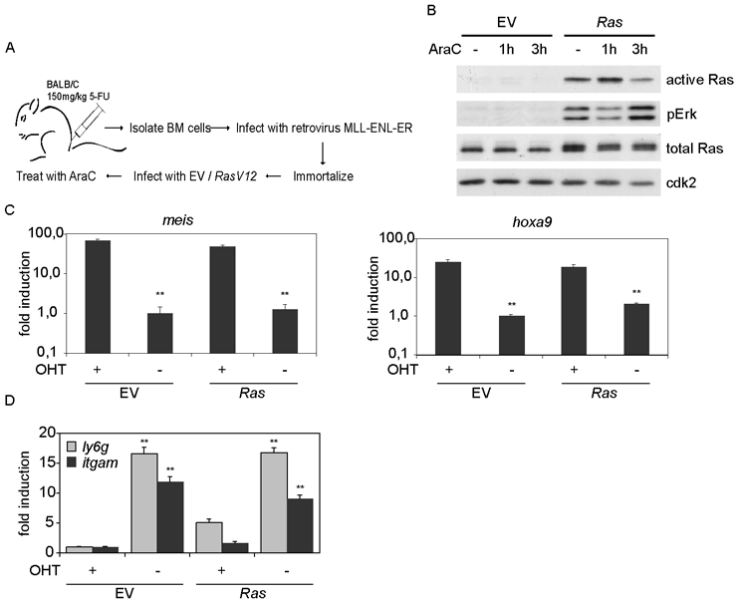
Generation of MLL-ENL-ER cells expressing oncogenic Ha-RasV12. (A) Flow chart of the generation of MLL-ENL-ER cells infected with either control retroviruses (empty vector; “EV cells”) or retroviruses expressing oncogenic *HA-RASV12* (generating *Ras* cells). (B) Elevated Ras expression and activity upon infection of MLL-ENL-ER cells. Control and *Ras* cells were treated with 50 µM cytarabine for the indicated times. Cell lysates were either incubated with GST-Raf bound to glutathione beads and immunoblots of bound proteins probed with pan-Ras antibody (“active Ras”) or cells lysates were probed with antibodies against phosphorylated Erk (pTyr-204) and Ras. Cdk2 served as loading control in this panel and all subsequent immunoblots. (C) MLL-ENL-ER target genes are not influenced by oncogenic *RAS*. Control and *Ras* cells were cultured for 12 days in the presence or absence of 4-OHT. Expression of *meis1* and *hoxa9* was analysed by RQ-PCR. Each column represents the mean±SD in this panel and all subsequent RQ-PCRs. The double asterisk represents statistical significance (p<0.01) of differences between cells cultured in the presence and absence of 4-OHT, respectively. (D) Oncogenic *RAS* does not abrogate the differentiation due to withdrawal of 4-OHT. Control and *Ras* cells were cultured for 12 days in the presence or absence of 4-OHT and the expression of the differentiation markers *ly6g* (encoding Gr1) and *itgam* (encoding Mac1) was measured by RQ-PCR. Statistical significance refers to differences between cells cultured in the presence or absence of 4-OHT, respectively.

We infected mouse bone marrow cells that had been immortalized with a conditional MLL-ENL-ER oncogene [27] with either control retroviruses (empty vector: “EV”) or retroviruses expressing an oncogenic Ha-RasV12 protein (generating EV and *Ras* cells; Figure 1A). Immunoblots confirmed that *Ras* cells expressed elevated levels of the Ha-Ras protein and displayed activation of Ras proteins as determined by binding to a GST-Raf protein and phosphorylation of Erk, a downstream effector of Ras (Figure 1B). Expression of *RAS* did not significantly affect the expression of MLL-ENL-ER (Figure S1) and of *meis1* and *hoxa9,* which are critical target genes of MLL-ENL [28] (Figure 1C). Furthermore, withdrawing 4-hydroxy-tamoxifen (OHT) to switch off the function of the MLL-ENL-ER chimera resulted in differentiation of both control (EV) and *Ras* cells as shown by the increased expression of markers of monocytic (*itgam* encoding Mac 1) and granulocytic (*ly6g* encoding the Gr1 antigen) differentiation (Figure 1D) and in downregulation of *meis1* and *hoxa9* expression (Figure 1C), demonstrating that oncogenic *RAS* did not influence the expression of these markers independent of MLL-ENL-ER.

**Figure 2:**
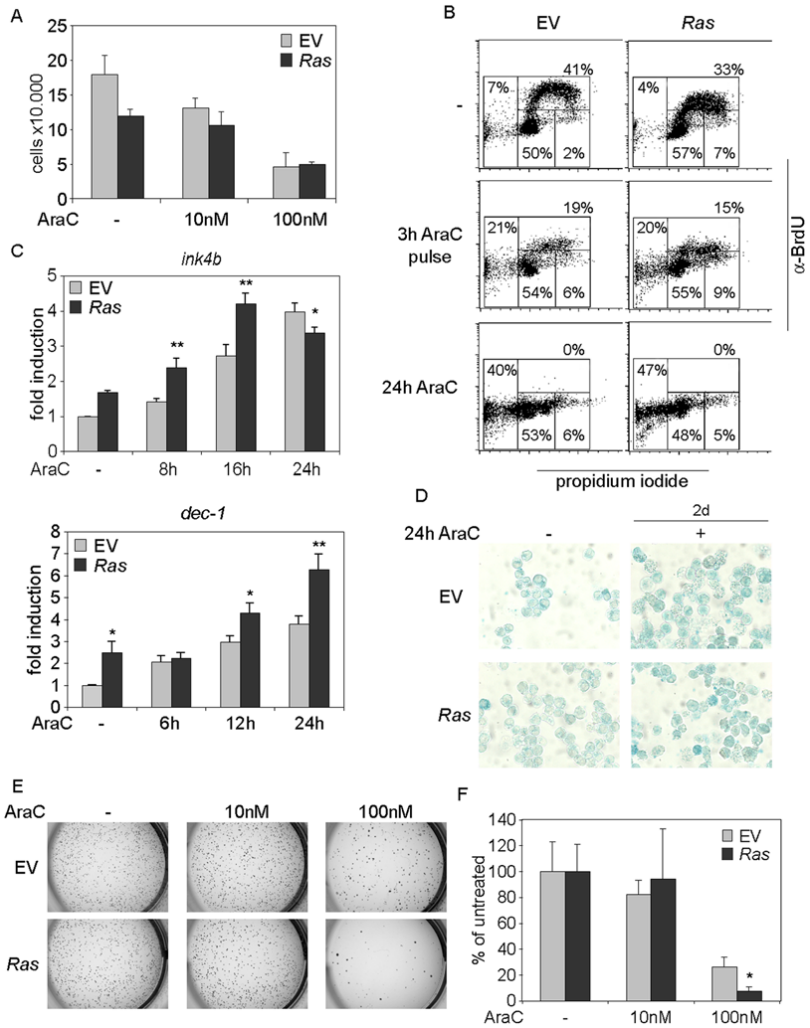
Oncogenic *RAS* compromises clonogenicity upon treatment with cytarabine. (A) Control and *Ras* cells show no difference in viability upon cytarabine (AraC) treatment. Cells were treated with cytarabine for 24 hours as indicated and live cells were counted in triplicates using exclusion of trypan blue as criterion. (B) Cytarabine has identical effects on replication and apoptosis of control and *Ras* cells, respectively. Control and *Ras* cells were treated either for three hours with 10 µM cytarabine and subsequently cultured for 21 hours in the absence of cytarabine or continuously treated for 24 hours. As a control, cells were cultured without drug. One hour before harvesting, BrdU was added to the culture. Cells were stained with propidium iodide and α-BrdU-FITC antibodies and subjected to FACS analysis. (C) Cytarabine induces expression of senescence markers *ink4b* and *dec-1* in control and *Ras* cells. Cells were cultured with 350 nM cytarabine for the indicated times and expression was analysed by RQ-PCR. At each time point statistical significance of differences between control cells and *Ras* cells was calculated. The asterisks in this panel and all subsequent panels represent statistical significance at p<0.05 for single asterisk and p<0.01 for double asterisk. (D) Senescence associated acidic β-galactosidase staining reveals no difference between control and *Ras* cells. Cells were treated with 350 nM cytarabine for 24 hours, then cultured for additional 48 hours in the absence of cytarabine and subjected to an acidic β-galactosidase assay. (E) *Ras* cells show compromised colony formation upon cytarabine treatment. Control and *Ras* cells were treated for 24 hours either with 10 nM or 100 nM cytarabine as indicated. Samples of 3.000 cells each were plated in methylcellulose. Colonies were stained after four days. (F) Quantification of (E). The graph shows the number of colonies relative to the number of colonies observed in the absence of cytarabine. The graph shows the average of three independent experiments.

Treatment with cytarabine (AraC) had no significant effect on expression of total or active Ras in either control or *Ras* cells (Figure 1B). In response to treatment with either low (10 nM) or elevated (100 nM) concentrations of cytarabine, both control and *Ras* cells showed a comparable decrease in cell number (Figure 2A). Furthermore, cytarabine inhibited DNA replication in both cell types to a similar extent, as measured by BrdU incorporation in a two-dimensional FACS analysis (Figure 2B). This analysis also showed that both cell types underwent apoptosis in response to either a transient (three hour pulse) or prolonged treatment (24 hours) with cytarabine (detected as cells with a subG1 DNA content in Figure 2B). Addition of cytarabine led to an increase in expression of two marker genes of senescence, *ink4b* and *dec-1* [29], in both control and *Ras* cells; possibly, therefore, cytarabine can induce senescence in MLL-ENL cells (Figure 2C). However, the cells were only weakly positive for a second marker of senescence, acidic β-galactosidase, and this was not affected by cytarabine (Figure 2D), suggesting that induction of senescence does not account for the selective loss of clonogenicity observed in *Ras* cells (see below).

### *Ras* cells show reduced colony formation potential and enhanced checkpoint activation after treatment with cytarabine

We next tested the ability of control and *Ras* cells to form colonies in semisolid medium after treatment with cytarabine. Cells were pre-treated in suspension for 24 hours with cytarabine at different concentrations, then plated in semisolid medium in the absence of the drug. Colony formation was scored after four days. No apparent difference was found between the two cell types in the absence or in the presence of lower doses of cytarabine (10 nM) (Figure 2E); pre-incubation with a higher concentration of 100 nM cytarabine led to a moderate (3.5fold) decrease in the colony forming capacity of control cells; in contrast, the same treatment largely abolished the clonogenic potential of *Ras* cells (Figures 2E,F). Thus, transient exposure to cytarabine reduces the clonogenic potential of *Ras* cells more strongly than that of control cells (Figure S2A). Slightly elevated concentrations of cytarabine were required to suppress colony formation in a second, independently derived clone of MLL-ENL cells (Figure S2B). Importantly, expression of *RAS* also enhanced the sensitivity to cytarabine in these cells, confirming that the difference in sensitivity was due to expression of *RAS* and not due to variations during the infection and selection of cells in culture (Figure S2B).

Since we observed no difference between control and *Ras* cells in the total level of apoptosis or senescence upon exposure to cytarabine, we considered two mutually non-exclusive possibilities to account for the differences observed in the clonogenic assays: First, MLL-ENL and *Ras* cells – despite being clonal isolates – might be heterogeneous with respect to clonogenic potential. Therefore, the response to cytarabine that is measured in the total cell pool may be determined by the majority of non-clonogenic cells and may not reflect the response of a potentially small pool of clonogenic cells; consistent with these suggestion, we observed that only a fraction of both cell types expressed the monocyte/macrophage marker Mac1 or c-kit, which is a marker of stem and progenitor cells (Figure S3A,B) and that the clonogenic population of both control and *Ras* cells could be enriched by isolating either a Mac1-depleted or a c-kit-enriched population (Figure S3C). Therefore, in both control and *Ras* cells, the cells enriched for a more immature phentype revealed similar colony forming capacity, arguing that they did not express a qualitative difference with regard to colony formation. Second, the clonogenic potential might be reduced by a process that is distinct from both apoptosis or senescence; this possibility was addressed in experiments described further below.

**Figure 3:**
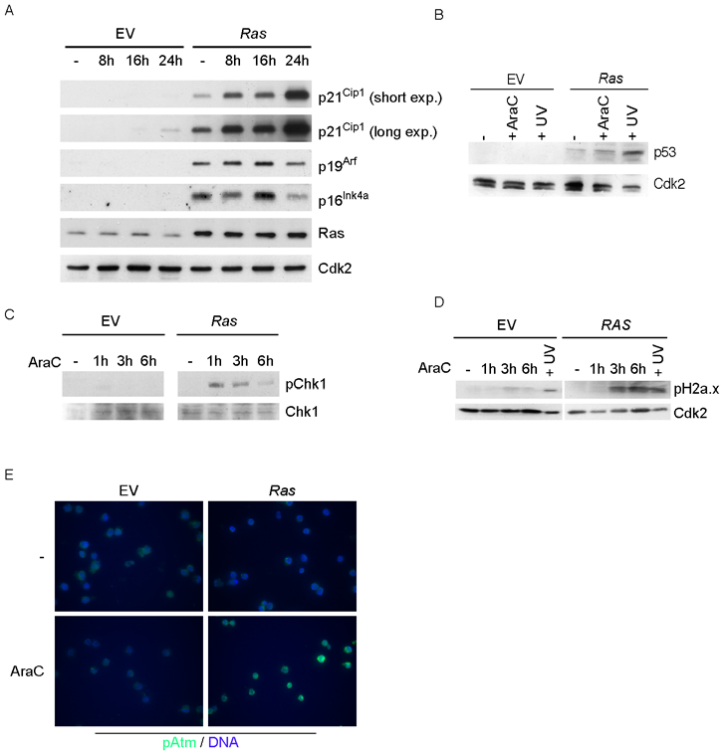
*Ras* cells express elevated levels of p16^Ink4a^ and p19^Arf^ and show increased checkpoint activity. (A) Immunoblot documenting the elevated basal expression and further induction of p21^Cip1^ after cytarabine treatment and increased expression of p16^Ink4a^ and p19^Arf^ in *Ras* cells. Control and *Ras* cells were treated with 350 nM cytarabine for the indicated times and cell lysates were probed with the indicated antibodies. Cdk2 is shown as loading control. (B) *Ras* cells express elevated levels of p53. Control and *Ras* cells were either treated with 100 µM cytarabine for five hours or exposed to UVB for 3.5 min and subsequently incubated for five additional hours. Immunoblots of cell lysates were probed with antibodies against p53 and Cdk2 as shown. (C) Phosphorylation of Chk1 is increased in *Ras* cells after treatment with cytarabine. Control and *Ras* cells were treated with 100 µM cytarabine for the indicated times. The panels show immunoblots of cell lysates subjected to antibodies against Chk1 and phospho-Chk1 (pSer345). (D/E) *Ras* cells show elevated checkpoint activation upon cytarabine treatment. Control and *Ras* cells were treated as before. Panel D shows immunoblots of cell lysates that were probed with antibodies directed against phospho-H2a.x (pSer139) and Cdk2 (loading control). Panel E shows immunofluorescence pictures of control and *Ras* cells treated with 100 µM cytarabine for one hour and stained with antibodies directed against phosphorylated Atm (pSer1981) (green). DNA was counterstained with Hoechst (blue).

To identify the mechanisms underlying the impaired clonogenicity of *Ras* cells, we wondered whether either of two events that occur in response to oncogenic activation of Ras could account for this observation: first, oncogenic Ras induces the expression of p16^Ink4a^ and p19^Arf^, leading to increased levels of p53, and p21^Cip1^ [19], [30]. Consistent with these observations, *Ras* cells expressed elevated levels of p16^Ink4a^ and p19^Arf^, and this was independent of the exposure to the DNA-damaging agent cytarabine; consequently, levels of p21^Cip1^ and p53 were elevated in *Ras* cells even in the absence of cytarabine (Figures 3A,B). Second, oncogenic Ras can induce a DNA damage response in primary cells, which leads to activation of the Atm and Atr checkpoint kinases [24], [25], [31]. We did not observe elevated levels of phosphorylated Chk1 or H2a.x, which are targets of the Atm or Atr kinases in immunoblots of either control or *Ras* cells before treatment with cytarabine (Figures 3C,D); furthermore, immunofluorescence did not reveal a difference in phosphorylated Atm in untreated cells (Figure 3E). However, cytarabine strongly induced phosphorylation of Chk1 in *Ras* cells, whereas the phosphorylation of this protein was hardly detectable in control cells treated with cytarabine. Unphosphorylated Chk1 was not differentially expressed between control and *Ras* cells (Figure 3C). This difference in checkpoint responses was not due to a reduced rate of DNA replication in control cells, which would lead to a reduced incorporation of cytarabine (Figure 2B). *Ras* cells also displayed strongly elevated levels of phosphorylated H2a.x and Atm after incubation with cytarabine relative to control cells (Figures 3D,E). Consistent with these observations, exposure to cytarabine led to a further increase in levels of p21^Cip1^ in *Ras* cells, whereas induction of p21^Cip1^ was hardly detectable in control cells (Figure 3A). The data show that oncogenic *RAS* cooperates with cytarabine in activation of DNA damage-dependent checkpoints.

Both mutation and enhanced expression of *RAS* genes have been reported in AML [32]. To determine whether elevation of checkpoint responses is specific for oncogenic Ras or whether it reflects the elevated levels of total Ras present in *Ras* cells, we determined the level of Chk1 phosphorylation in two pools of MLL-ENL cells that had been infected with retroviruses expressing wild type *Ha-RAS* (Figure S4). Enhanced phosphorylation of Chk1 was observed in cells expressing mutated *RAS*, but also – albeit to a significantly lesser extend - in cells expressing high-levels of wild type *RAS*. Most likely, therefore, both, the oncogenic mutation of *RAS* and - more weakly – the enhanced expression contribute to the altered checkpoint activity of *Ras* cells.

### DNA-damaging agents such as daunorubicine and etoposide also reveal differential effects in *Ras* cells

**Figure 4:**
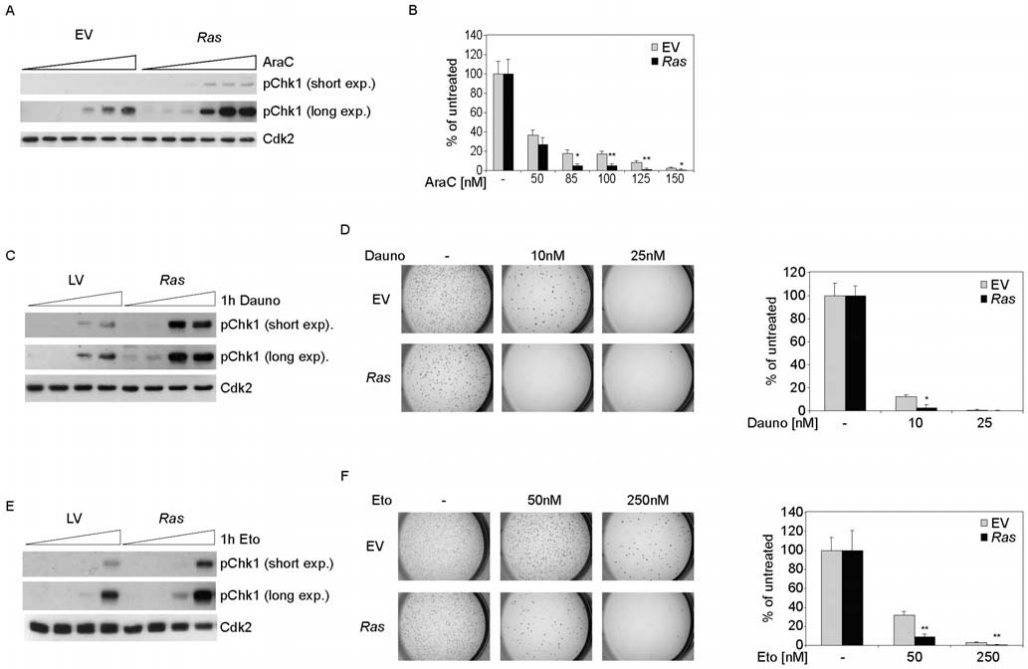
*Ras* cells show elevated checkpoint activity in response to treatment with cytarabine, daunorubicine or etoposide. (A) Phosphorylation of Chk1 in response to cytarabine treatment is dose-dependent and elevated in *Ras* cells. Control and *Ras* cells were treated with 350 nM, 1 µM, 10 µM, 50 µM and 100 µM cytarabine for 1 hour and analyzed by immunoblot probing for phospho-Chk1 (pSer345) and Cdk2. (B) Control and *Ras* cells show dose-dependent decrease of colony formation upon cytarabine treatment. Samples of 3.000 cells each were plated in methylcellulose. Shown is the average number of colonies observed in three independent assays as percentage of colonies observed in control incubation in the absence of cytarabine. *Ras* cells show significantly fewer colonies compared to control cells (85 nM, 150 nM: p<0.05; 100 nM, 125 nM: p<0.01). (C) Daunorubicine leads to enhanced phosphorylation of Chk1 in *Ras* cells. The immunoblot shows lysates of cells, which were treated with 1 µM, 10 µM and 50 µM daunorubicine for 1 hour and probed as before. (D) *Ras* cells treated with daunorubicine show compromised colony formation. Control and *Ras* cells were incubated for 24 hours with the indicated concentrations of daunorubicine. 3.000 cells each were plated in methylcellulose and stained after four days. The graph shows the average number of colonies observed in three independent experiments relative to the number of colonies observed in the absence of drug. Statistical significance refers to differences between the colony formation of control cells and *Ras* cells. (E) *Ras* cells are more sensitive in checkpoint activation towards etoposide treatment. Cells were treated with 1 µM, 10 µM and 50 µM etoposide for 1 hour and lysates were analysed by immunoblot using the same antibodies as before. (F) Etoposide has a stronger effect on the colony formation of *Ras* cells. Control- and *Ras* cells were either untreated or treated with 50 nM and 250 nM etoposide for 24 hours, respectively, before 3.000 cells each were plated in methylcellulose. The graph shows the average number of colonies observed in three independent experiments relative to the number of colonies observed in the absence of drug.

In order to elucidate if the observed effects on DNA-damage checkpoints and the impaired capacity to form colonies *in vitro* were specific for cytarabine, we investigated the effects of the topoisomerase II inhibitors daunorubicine and etoposide on colony formation and DNA-damage checkpoints in control and *Ras* cells and compared this to cytarabine. Both control and *Ras* cells showed a dose-dependent phosphorylation of Chk1 (Figure 4A) and inhibition of colony formation (Figure 4B) in response to cytarabine. Consistent with our previous data, *Ras* cells showed an enhanced checkpoint response at all cytarabine concentrations tested (for a quantitation, see Figure S5A) and were more sensitive with respect to colony formation (Figure S5B) at elevated concentrations of cytarabine. Similarly, when control and *Ras* cells were treated with daunorubicine and etoposide, *Ras* cells appeared more sensitive towards incubation with these drugs with regard to phosphorylation of Chk1 and colony formation, respectively (Figures 4C and D: daunorubicine; 4E and F: etoposide). Thus, the differential response of *Ras* vs. control cells was not only seen after treatment with cytarabine, but also with daunorubicine and etoposide.

### Oncogenic *RAS* induces myeloid differentiation after cytarabine treatment

**Figure 5:**
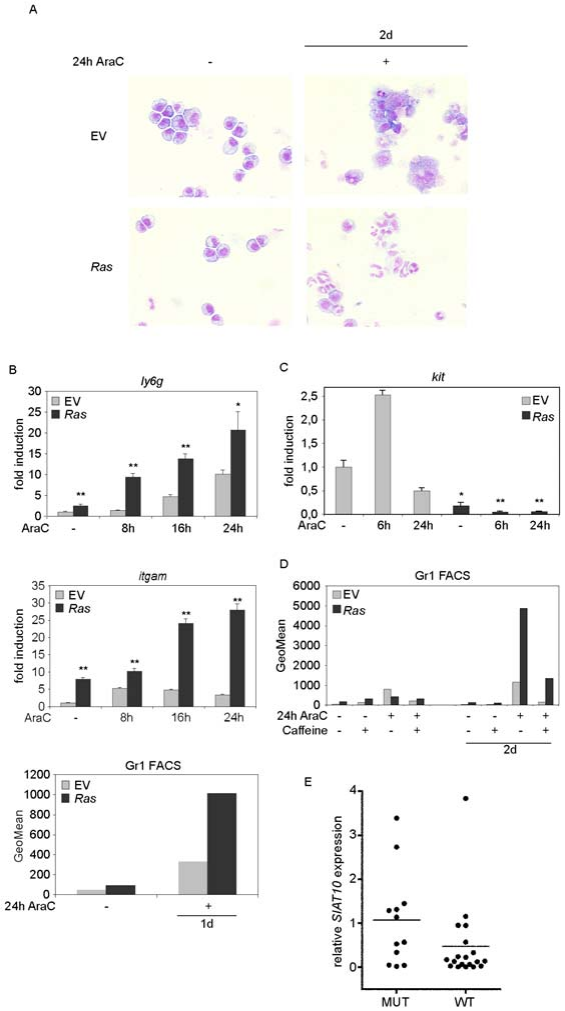
*Ras* cells show myeloid differentiation upon cytarabine treatment. (A) Morphological analysis reveals differentiation of *Ras* cells. Control and *Ras* cells were treated with 350 nM cytarabine for 24 hours. Cells were washed and cultured for additional 48 hours in the absence of cytarabine and stained with May-Grunwald/Giemsa. Untreated control and *Ras* cells have a similar blastic phenotype. Treatment with cytarabine induces a modest differentiation in control cells; this is potentiated in *Ras* cells. Shown is a representative experiment. Specific differential counts of these cells is presented in Figure S6A. (B) Upper panels: Elevated expression of markers of differentiation in *Ras* cells. Control and *Ras* cells were cultured with 350 nM cytarabine for the indicated times and *ly6g* and *itgam* mRNAs were analysed by RQ-PCR. The asterisk represents statistical significance of differences between control- and *Ras* cells at the indicated time points. Lower panel: Gr1 protein expression is elevated in *Ras* cells. Control and *Ras* cells were treated with 350 nM cytarabine for 24 hours washed and cultured for additional 24 hours in the absence of cytarabine. Cells were harvested, stained with α-Gr1-PE antibody and subjected to FACS analysis. Shown is a representative experiment. (C) *Ras* cells show decreased expression of stem cell factor *kit.* Control and *Ras* cells were cultured as before and RQ-PCR was performed. The asterisk represents statistical significance of differences between control- and *Ras* cells at the indicated time points. (D) The Atm/r-kinase inhibitor caffeine abrogates the cytarabine-induced differentiation in *Ras* cells. Control and *Ras* cells were treated with either 100 nM cytarabine or 0.5 mM caffeine, respectively, or with both components simultaneously for 24 hours, washed and cultured for additional 48 hours in the absence of cytarabine. Cells were harvested, stained with α-Gr1-PE antibodies and subjected to FACS analysis. Shown is a representative experiment. (E) Primary AML cases with oncogenic *N-RAS* mutations reveal a higher expression of the myeloid differentiation marker *SIAT10* upon RQ-PCR analysis. 12 cases with (group 1), and 19 cases without (group 2) oncogenic *N-RAS* mutations were analyzed for *SIAT10* expression relative to HL60 cells. Shown are the individual samples and the mean expression (p = 0.04).

We next asked whether the selective loss of clonogenic potential of *Ras* cells in response to cytarabine correlates with an increase in differentiation [14]–[18], [33]. Control and *Ras* cells were incubated with cytarabine, and differentiation was determined by analyzing cellular morphology and the expression of markers of granulocytic (Gr1) and monocytic differentiation (Mac1). There was a significant enhancement of differentiation in cells expressing oncogenic *RAS* after treatment with cytarabine as observed in May-Grunwald-Giemsa-stained slides (Figure 5A and Figure S6A) as well as in RQ-PCR and FACS analyses of *ly6g* (encoding the Gr1 antigen) and *itgam* (encoding Mac 1) (Figure 5B and Figure S6B). Consistent with these observations, the mRNA levels of the stem cell marker *kit* were lower in *Ras* cells and expression became virtually undetectable upon treatment with cytarabine (Figure 5C).

To test whether the enhanced differentiation observed in *Ras* cells depends on the elevated checkpoint activity in response to cytarabine, we incubated control and *Ras* cells for 24 hours with cytarabine in the presence or absence of caffeine, an inhibitor of the Atm and Atr kinases [34]. Initial experiments showed that incubation with 0.5 mM caffeine was sufficient to inhibit phosphorylation and thus activation of Chk1, as demonstrated by the reduced level of p53 (Figure S7A+B). Notably, treatment with caffeine together with cytarabine markedly decreased the myeloid differentiation of *Ras* cells (Figure 5D). In contrast, addition of caffeine did not restore the clonogenic potential of *Ras* cells treated with cytarabine, potentially because caffeine showed significant toxicity in long-term experiments (not shown). Taken together, the data show that the cytarabine-induced loss of clonogenicity correlates with a checkpoint-dependent induction of cellular differentiation of *Ras* cells.

Importantly, inducing cellular differentiation by withdrawal of 4-hydroxy-tamoxifen and subsequent inactivation of the MLL-ENL-ER chimeric protein did not elevate checkpoint activation in the absence of oncogenic *RAS* (Figure S8), suggesting that the effects of oncogenic Ras on checkpoint activity are upstream and independent of its effects on cellular differentiation.

Figures 5B and C show a higher spontaneous differentiation of *Ras* cells, even without *in vitro* treatment with cytarabine. In order to investigate if oncogenic *Ras* induced myeloid differentiation, which is potentiated by activating DNA-damaging agents such as cytarabine, may also be observed in primary AML, we analyzed primary AML samples using cDNA expression analysis. We took advantage of primary AML cases diagnosed within the AML-SHG Germany multicenter study group and selected 31 AML cases with inversion (16) to have a comparable genetic background. AML with oncogenic *N-RAS* mutations (N = 12) revealed a different expression signature as compared to AML lacking such mutations (N = 19). A Gene Set Enrichment Analysis demonstrated that genes characteristic for hematopoietic progenitors were expressed at higher levels in samples harbouring wild-type *N-RAS* while genes associated with mature blood cells rather than progenitor and stem cell compartments were strongly enriched (up-regulated) in the *N-RAS* mutant samples (not shown) [35], [36]. We confirmed the more differentiated phenotype of *N-RAS* mutant AML using RQ-PCR for the *SIAT10* gene, which is known to be progressively up-regulated during myeloid differentiation (Figure 5E) [35]. The higher spontaneous differentiation observed for *Ras* cells *in vitro* was therefore also detected in primary AML samples.

### Expression of p16^Ink4a^-resistant Cdk4R24C does not abrogate myeloid differentiation

Relative to control cells, *Ras* cells express elevated levels of p16^Ink4a^ (Figure 3A), which inhibits Cdk4 kinase activity, leading to activation of the retinoblastoma tumour suppressor protein (Rb). In addition, they express elevated levels of p19^Arf^ and show enhanced checkpoint activity, both leading to activation of p53. To identify which if any of these two proteins is a critical downstream effector of Ras that mediates the cytarabine-induced loss of clonogenicity, we generated *Ras* cells that express either Cdk4R24C, a melanoma-derived mutant of Cdk4 that is resistant to inhibition by p16^Ink4a^ [37] or a dominant-negative allele of p53 [38].

Immunoblots revealed that Cdk4R24C was expressed in *Ras* cells and had no effect on the activation of Chk1 in response to treatment with cytarabine (Figure S9A); furthermore, expression of Cdk4R24C led to elevated levels of phosphorylated pRb, consistent with its ability to negate the effect of p16^Ink4a^ (Figure S9B). Importantly, expression of Cdk4R24C had no effect of the cytarabine-induced differentiation of *Ras* cells (Figures S9C,D,E) and further suppressed their clonogenic potential in the presence of cytarabine (Figure S9F), strongly suggesting that p16^Ink4a^ is not a critical mediator of Ras action in this setting.

### Dominant negative p53 inhibits oncogenic *RAS*- and cytarabine-induced differentiation

**Figure 6:**
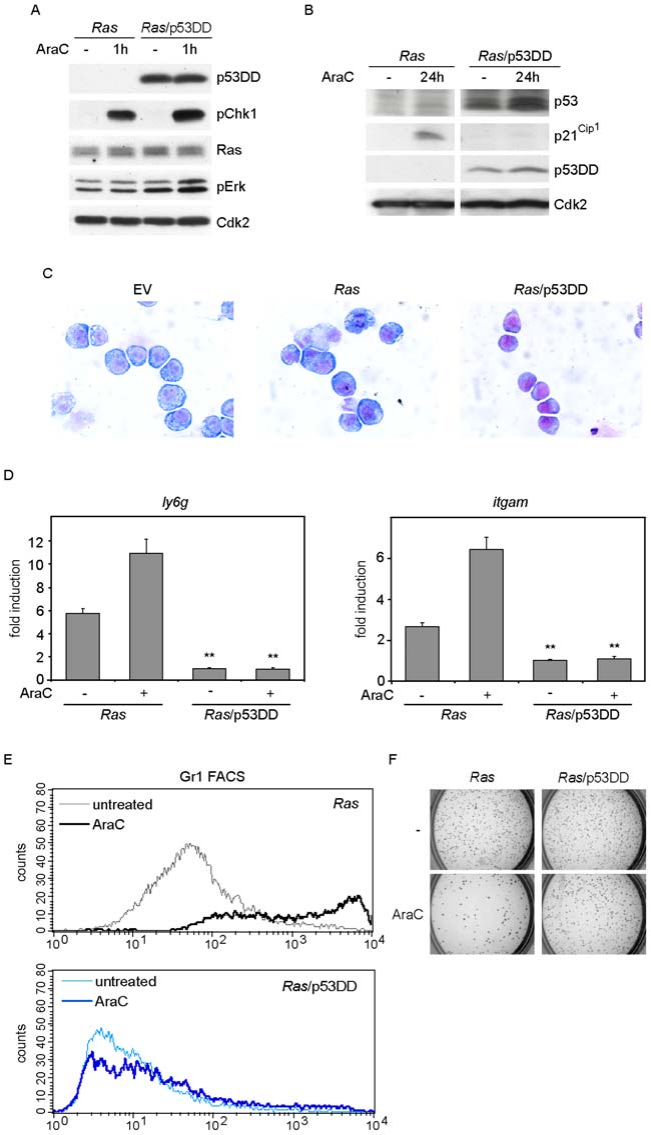
p53 is required for *RAS*- and cytarabine-induced suppression of clonogenicity. (A) Phosphorylation of Chk1 is independent of functional p53. *Ras* and *Ras*/p53DD cells, expressing dominant negative p53, were treated with 100 µM cytarabine for one hour. The panels show immunoblots probed with the indicated antibodies. (B) Cytarabine-induced expression of p21^Cip1^ is abrogated by p53DD. *Ras* and *Ras*/p53DD cells were incubated with 350nM cytarabine for 24 hours. Immunoblots of cell lysates were probed with antibodies against p53, p53DD, p21^Cip1^ and Cdk2. (C) p53DD induces a blast-like morphology in *Ras* cells. Cells were stained with May-Grunwald/Giemsa and pictures were taken as described before. (D) p53DD suppresses expression of *ly6g* and *itgam* mRNA in *Ras* cells. *Ras* and *Ras*/p53DD cells were treated with 350 nM cytarabine for 24 hours and RQ-PCR was performed as before. The double asterisk represents statistical significance of differences between *Ras* cells and *Ras*/p53DD cells (p<0.01). (E) Expression of Gr1 proteins is suppressed by p53DD. *Ras* and *Ras*/p53DD cells were treated with 350 nM cytarabine for 24 hours and subsequently cultured for additional two days in the absence of cytarabine. Cells were subjected to FACS analysis using α−Gr1-PE antibodies. (F) Expression of p53DD enhances the colony formation potential of *Ras* cells upon cytarabine treatment. The assay was performed as described in Figure 2.

To test whether the observed differentiation depends on p53, we expressed a dominant negative allele of p53 (p53DD) in *Ras* cells (generating *Ras*/p53DD cells) [38]. Immunoblots confirmed the expression of p53DD in these cells, and showed that cytarabine-induced phosphorylation of Chk1 is independent of p53 (Figure 6A). In contrast, expression of p53DD abrogated cytarabine-induced p21^Cip1^ expression, a downstream target of p53; furthermore levels of endogenous p53 were elevated in cells expressing p53DD, indicative of downregulation of the *MDM2* gene (Figure 6B and Figure S10). These data demonstrate that p53DD blocked p53 function in *Ras* cells.

Importantly, May-Grunwald-Giemsa staining showed that untreated *Ras*/p53DD cells had an immature phenotype in contrast to *Ras* cells, which showed a monocytic/macrophage like morphology (Figure 6C). RQ-PCR analysis showed that expression of p53DD decreased the basal expression of *ly6g* and *itgam* mRNAs and abrogated the cytarabine-induced increase in expression of these markers of myeloid differentiation (Figure 6D); very similar results were obtained in a FACS analysis of Gr1 and Mac1 protein expression (Figure 6E; Figure S11). In order to analyse whether the Ras-induced inhibition of colony growth was also mediated by p53, *Ras*/p53DD cells were analysed in the colony test after pre-treatment with cytarabine as before. Importantly, expression of p53DD, but not the inactive L344P mutant of p53DD, restored the clonogenic potential of *Ras* cells upon transient exposure to cytarabine (Figure 6F and Figure S12). Similar results were obtained by inhibition of p53 with the small molecule pifithrin-α [39] (Figure S12).

Taken together these data strongly suggest that the loss of clonogenicity and increased differentiation observed in *Ras* cells in response to cytarabine is mediated by activation of p53.

### Nutlin-3 enhances the biological effects induced by oncogenic *RAS* and cytarabine

As shown above, p53 is needed for the cytarabine and oncogenic *RAS* driven myeloid differentiation program in leukemia cells. Nutlin-3 activates endogenous p53, as it inhibits the Mdm2-induced degradation of p53 [40]. Nutlin-3 can induce p53-dependent apoptosis in acute leukemia cells [41]–[43]. Interestingly, a nutlin-dependent maturation program has recently been described in AML cells [44]. We therefore wanted to know whether incubation of *Ras* cells with cytarabine together with nutlin-3 lead to increased myeloid differentiation in these cells.

**Figure 7:**
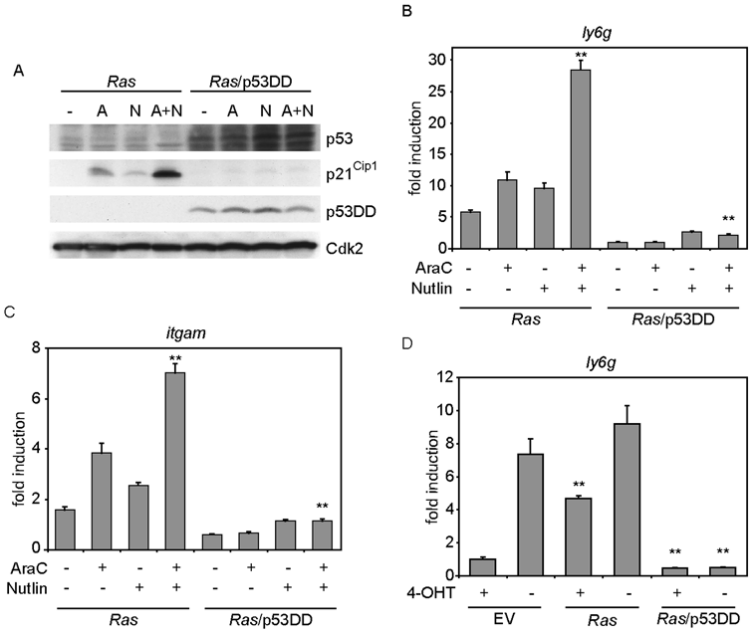
Nutlin-3 enhances the biological effects of oncogenic *RAS* in a p53-dependent manner. (A) Nutlin-3 and cytarabine co-operate to induce p21^Cip1^ expression in *Ras* cells but not in *Ras*/p53DD cells. Cells were treated for 24 hours with either 350 nM cytarabine (A) or nutlin-3 (N) alone or simultaneously (A+N) as indicated. The immunoblots show the expression of p53, p21^Cip1^ and p53DD in the indicated cell types. (B, C) p53DD abrogates the nutlin- and cytarabine-induced expression of *ly6g* (B) and *itgam* (C) mRNAs. *Ras*- and *Ras*/p53DD-cells were treated for 24 hours with either 350 nM cytarabine or nutlin-3 alone or simultaneously as indicated and RQ-PCR was performed. The double asterisk indicates a significant difference between *Ras* cell untreated and treated with cytarabine and nutlin-3 and between *Ras* cells and *Ras*/p53DD cells in the presence of cytarabine and nutlin (p<0.01). (D) Differentiation of *Ras* cells due to withdrawal of 4-OHT is blocked by p53DD. Control, *Ras* and *Ras*/p53DD-cells were cultured for 12 days in the presence or absence of 4-OHT and the expression of *ly6g* mRNA was analysed by RQ-PCR. The double asterisk represents statistical significance (p<0.01) of differences between control cells and *Ras* cells cultured in the presence of 4-OHT as well as differences between *Ras* cells and *Ras*/p53DD cells cultured in the presence and absence of 4-OHT, respectively.

To address whether the biological effects of endogenous p53 could be enhanced using nutlin-3, we measured the expression of p21^Cip1^ as a downstream effector of p53. Expectedly, expression of p21^Cip1^ increased after incubation of *Ras* cells with cytarabine (Figure 7A). Nutlin-3 incubation also induced, to a lesser degree, p21^Cip1^ expression. Importantly, co-incubation of nutlin-3 together with cytarabine potentiated cytarabine-induced p21^Cip1^ expression in *Ras* cells (Figure 7A); in contrast, neither cytarabine nor nutlin-3 had any effect on p21^Cip1^ expression in *Ras*/p53DD cells.

We next asked whether nutlin-3 was also able to enhance cellular differentiation in *Ras* cells. RQ-PCR analysis showed that expression of differentiation marker genes *ly6g* and *itgam* was significantly increased when *Ras* cells were co-incubated with nutlin-3 and cytarabine (Figures 7B,C). Moreover, nutlin-3 was unable to stimulate differentiation of *Ras/*p53DD cells, demonstrating that it does not act via a p53-independent mechanism. Together, the data show that Mdm2-mediated degradation of p53 limits the differentiation observed in response to cytarabine and that combination of nutlin-3 enhances the differentiation-inducing effect of cytarabine.

Finally, we noted that withdrawal of 4-hydroxy-tamoxifen to switch off the MLL-ENL-ER chimera induced expression of *ly6g* and *itgam* in control and in *Ras* cells, but was unable to do so in *Ras*/p53DD cells (Figure 7D), arguing that p53 is essential for granulocytic and monocytic differentiation, at least in the context of oncogenic Ras.

## Discussion

Oncogenic *RAS* mutations are among the most frequent mutations observed in human cancers (reviewed in [6]). Until recently, the prognostic role of oncogenic *RAS* in AML was not well understood. Some studies reported a correlation with poor outcome [45]–[47], whereas others observed a better prognosis in AML with oncogenic *RAS* mutations [48]–[50]. We have recently extended these analyses and have demonstrated an interaction between oncogenic *RAS* and the dose of cytarabine used during postinduction-treatment with respect to the cumulative incidence of relapse and overall survival [26]. Patients, whose AML blasts revealed oncogenic *RAS* mutations and who had been treated with low-dose cytarabine, had the worst prognosis (highest incidence of relapse; worst survival) [26]. In contrast, those with oncogenic *RAS* randomly treated with high-dose cytarabine had the best prognosis of all groups (lowest incidence of relapse, best survival). Patients with wild type *RAS* had only little benefit from cytarabine dose-escalation, and their prognosis was in between the patients with *RAS* mutations. The molecular basis for this observation remained unclear.

In order to molecularly understand the interaction of oncogenic *RAS* with cytarabine in AML, we took advantage of mouse bone marrow cells that had been immortalized using a conditional MLL-ENL-ER oncogene [27], [28] and that were co-infected with either an empty vector (control cells) or a vector expressing oncogenic *RAS* (*Ras* cells). We chose leukemic cells expressing MLL-ENL for several reasons: first, MLL-ENL is a potent oncogene and is able to transform and immortalize progenitor cells at various levels of myeloid differentiation, although high-doses of growth factors are still needed for *in vitro* culture, most likely to substitute for a lacking class I mutation in these cells [27], [51]. Second, MLL-ENL expressing progenitor cells define a leukemia-initiating cell population resembling acute myeloid leukemia in humans [52]; third, once MLL-ENL-ER is switched off, the cells differentiate and undergo apoptosis, demonstrating the importance of this fusion gene for maintaining self renewal and growth.

Although *in vivo* therapy with cytarabine underlies a complex pharmacodynamic and pharmacokinetic regulation which may not be reproduced in the cell culture system used in this study [53], the MLL-ENL cells showed phenomena *in vitro* which resemble the observations made in the clinical study [26]. Notably, there was no significant difference between the cell number, cell survival or apoptosis of *Ras* and control cells treated with cytarabine. This corresponds with the clinical situation where a significant difference between AML-patients with and without *RAS* mutations with regard to complete remission was not found. Instead, the clinical observation that patients with oncogenic *RAS* relapse less frequently after complete remission suggested that the number of clonogenic stem cells able to cause AML relapse may be significantly decreased in AML with oncogenic *RAS* mutations upon treatment [26]. This may correlate with the lower *in vitro* clonogenicity of *Ras* cells that have been transiently exposed to cytarabine (and, as shown in Figure 4, to other cytotoxic drugs such as daunorubicine and etoposide).

Our analysis provides several molecular correlates for these observations and suggests that the difference in response to cytarabine is due to known biological properties of oncogenic *RAS*. Notably, Ras is known to activate expression of several proteins associated with cellular senescence, such as p16^Ink4a^, p19^Arf^ and p21^Cip1^ [20] and we confirmed these observations for the leukemic cells studied here (Figure 3A). Oncogenic *RAS* also activates a DNA damage response in primary fibroblasts [24], [25], [54]. While oncogenic *RAS* was unable to induce a DNA-damage response in MLL-ENL cells by itself, it strongly enhanced the DNA-damage response observed after incubation with DNA-damaging agents such as cytarabine. As a result, high levels of p53 are present in *Ras* cells treated with cytarabine (Figure 3B) and mediate their loss of clonogenicity (Figure 6F).

### Oncogenic *RAS* synergizes with cytarabine to enhance differentiation of AML cells

Activated DNA damage checkpoint frequently induce cell cycle arrest or apoptosis, yet we have not observed any difference in regard to apoptosis or proliferation after cytarabine treatment between *Ras* and control cells. It has been reported that cytarabine can induce myeloid differentiation (e.g. [33], [55]). Numerous studies have also shown that oncogenic *RAS* also induces differentiation [14]–[18]. We observed a very moderate induction of differentiation in control cells, when treated with cytarabine. This was significantly increased in *Ras* cells incubated with cytarabine (Figure 5). Therefore, either molecular change alone was ineffective in inducing full differentiation, whereas the combination of both did. Notably, the increased differentiation was abolished by incubation with caffeine, an inhibitor of the Atm and Atr kinases, demonstrating that it depends on checkpoint activation (Figure 5D). Taken together, the data suggest that oncogenic *RAS* in combination with higher doses of cytarabine induces differentiation *in vitro* and strongly decreases the clonogenic potential. Supporting this notion, *Ras* cells treated with cytarabine were much less likely to express the stem cell marker *kit* as compared to control cells (Figure 5C). Also, primary AML cells with oncogenic *N-RAS* mutations revealed a higher expression of differentiation markers as compared to patients lacking such mutations (Figure 5E).

### Oncogenic *RAS*- and cytarabine-induced myeloid differentiation depends on p53

*TP53* is frequently mutated in human cancer, but rarely in myeloid leukemias; therefore, the MLL-ENL transformed cells here mimic the p53 status found in AML. Importantly, our data identify p53 as a critical mediator of the enhanced differentiation and loss of clonogenicity observed in *Ras* cells upon exposure to cytarabine. This was supported by the observation that *Ras* cells expressing p53DD did not undergo differentiation upon treatment with cytarabine (Figures 6D,E). Also, treatment of *Ras* cells with both cytarabine and nutlin-3, an inhibitor of Mdm-2 induced degradation of p53, caused a further increase in p21^Cip1^ expression, which was paralleled by increased expression of differentiation markers (Figures 7A,B,C) and is consistent with a recent report that nutlin-3 can cause maturation in AML cells [44].

Notably, *Ras* cells expressing p53DD did not differentiate even when the MLL-ENL-ER oncogene was switched off by withdrawing 4-hydroxy-tamoxifen (Figure 7D), demonstrating that p53 is required for induction of differentiation of these cells. MLL-ENL has been shown to attenuate p53 function, since it antagonizes the interaction of p53 with the p300/CBP co-activator [56]; it is conceivable that this activity of MLL-ENL counteracts the induction of differentiation by low levels of active p53, such as might be present in cells expressing oncogenic *RAS* in the absence of cytarabine.

One *caveat* in our study is the use of *H-Ras*, while AML patients more frequently harbor mutations in the *N-RAS* or *K-RAS* genes [48]. However, the signaling pathways downstream of Ras proteins are conserved and *H-Ras* can substitute for *K-Ras* during embryonic development [57]. Furthermore, higher expression of differentiation markers in primary AML cells with oncogenic *N-RAS* mutations (Figure 5E) strongly suggests that mutant *H-Ras* and *N-Ras* function similarly to promote differentiation in hematopoietic cells.

Our findings may have implications for understanding how chemotherapeutic drugs exert their effects. Conventionally, cytostatic drugs such as cytarabine are thought to act as inhibitors of proliferation and inducers of apoptosis. This study suggests that the critical function of a cytotoxic drug such as cytarabine may be to promote the differentiation of tumor-initiating cells. That induction of differentiation is an attractive goal for anticancer therapy and is associated with higher cure rates has been demonstrated elegantly in acute promyelocytic leukemia, where high-doses of retinoic acid, given concomitantly with chemotherapy, overcomes the repressive effect of PML-RARα in differentiation. We propose that induction of differentiation should therefore be seen as a broader goal in AML therapy, e.g. for *in vitro* screening of new compounds, and in the development of new treatment protocols. The success of such a procedure will depend on the genetic background of the respective cancer cells.

## Materials and Methods

### Retroviral transduction of mouse primary hematopoietic cells

High-titer retrovirus supernatants were produced by transient transfection of the packaging cell line Phoenix-E using a standard Ca^2+^-phosphate precipitation method. Viral titers usually reached approximately 5×10^6^ CFU/ml. Retroviral transduction of primary hematopoietic cells was performed as described previously [58]. All constructs (Ha-RasV12, Cdk4R24C-Flag, p53DD (aa 302–390), p53LP (p53DD with L344P mutation) were cloned into pMSCV retroviral vectors (Clontech, USA) and cells were cultured in the presence of either puromycin, hygromycin or blasticidin, respectively. In total, we performed three independent infections of MLL-ENL cells with either control viruses or viruses encoding oncogenic *RAS*, using two different clones of MLL-ENL cells. The results we obtained were consistent between these independent experiments.

### Tissue culture and growth assays

Transduced bone marrow cells were kept either in MethoCult (M3234) methylcellulose medium (Cell systems, St. Katharinen, Germany) or in RPMI 1640 (Gibco, Karlsruhe, Germany). Recombinant mouse cytokines (Cell Systems, St. Katharinen, Germany) were added in the following concentrations: interleukin-3, interleukin-6, and granulocyte-macrophage colony-stimulating factor (GM-CSF), 10 ng/ml; SCF, 100 ng/ml. All liquid media were supplemented with 10% bovine fetal calf serum (Gibco, Karlsruhe, Germany) and penicillin-streptomycin. 4-Hydroxy-tamoxifen (4-OHT) was added to a final concentration of 100 nM from a 1 mM stock solution in ethanol.

To measure checkpoint responses in short-term assays, cells were treated either with up to 100 µM cytarabine (Merck, Darmstadt, Germany) for the indicated times or exposed to UV-B for 3.5 min and subsequently cultured at 37°C. Where indicated, cells were co-treated with 500 µM caffeine (Sigma Aldrich, Munich, Germany). For long-term assays, cells were exposed to 100 nM cytarabine unless indicated otherwise. For stabilization of p53 protein, Nutlin-3 (Sigma Aldrich, Munich, Germany) was added at a concentration of 5 µM. For inhibition of p53 protein cells were treated with 20 µM Pifithrin-α (Calbiochem, Gibbstown, NJ, USA).

For immunoblot analysis of phopho-Chk1, cells were exposed for 1 h to 1 µM, 10 µM and 50 µM daunorubicine (Pfizer, Berlin, Germany) and etoposide (Teva-Gry, Kirchzarten, Germany), respectively. Colony formation assays were performed with concentrations of 10 nM and 25 nM for daunorubicine and 50 nM and 250 nM for etoposide.

### Colony formation

The assays were performed in methylcellulose medium and colonies were stained with INT (Iodonitrotetrazolium chloride, Fluka, Buchs, Switzerland) at a final concentration of 1 mg/ml. For cell counting, methylcellulose was diluted in PBS, cells harvested and live cells were counted in triplicates of each sample using exclusion of trypan blue as criterion. Pictures of the colonies were captured using a Leica MZ125 binocular together with Leica DC300 camera and Leica IM1000 software (Leica Microsystems, Switzerland).

### Acidic β-galactosidase assay

Cells were assayed for the senescence-associated β-galactosidase activity by x-gal staining as described in [59] and subsequently transferred to slides by cytocentrifugation. Pictures were taken by Leica DMLB microscope with a Leica DFC420 camera and Leica DFC Twain software (Leica Microsystems, Switzerland).

### Morphological analysis

A cytocentrifuge was used to spin cells onto slides. Staining was performed using May-Grunwald and Giemsa (Sigma Aldrich, Munich, Germany) according to the manufacturers protocol.

### Immunofluorescence

Samples were fixed in 3,7% paraformaldehyde for 10 min, followed by permeabilization in PBS-T (PBS containing 0.2% Triton X-100). Nonspecific protein binding was blocked by 5% FCS (Gibco, Karlsruhe, Germany). Cells were stained using a primary antibody against phospho-Atm (pSer1981, Chemicon, USA) and a FITC-conjugated α-rabbit in parallel to Hoechst. Pictures were taken by BD Pathway 855 High-Content Bioimager with BD AttoVision 1.6 software (BD Biosciences, Heidelberg, Germany).

### Immunoblot and antibodies

Cells were lysed either in TNN buffer (50 mM Tris, pH 7.4, 150 mM NaCl, 0.5% NP-40, 5 mM EDTA, 1 mMDTT, protease and phosphatase inhibitors (Sigma Aldrich, Munich, Germany), Ripa buffer (10 mM NaPO_4_, pH 7.2, 150 mM NaCl, 1% NP-40, 0.1% Sodium Deoxycholate, 0.1% SDS) or NP-40-Lysisbuffer (50 mM Tris pH 8.0, 150 mM NaCl, 1% NP-40, pH 8.0). Proteins were separated by SDS gel electrophoresis and transferred to Immobilon-P (Millipore, CA, USA). Proteins were detected by immunoblotting. Antibodies used were phospho-Erk (pTyr204), Cdk2, p53, p21^Cip1^, p16^Ink4a^, Chk1, phospho-Rb (pSer795), Rb (all: Santa Cruz Biotechnology, Heidelberg, Germany), Ras (BD Transduction Laboratories), p19^Arf^ (Abcam, Cambridge, UK), phospho-Chk1 (pSer345), phospho-H2a.x (pSer139) (both: Cell signaling, USA) and phospho-Atm (pSer1981) (Chemicon, USA), p53DD (monoclonal mouse antibody pAb122) and Flag-antibody (Sigma Aldrich, Munich, Germany).

### Ras pull-down assay

The activity of Ras was analyzed with Ras Activation Assay Kit from Millipore (CA, USA) according to the manufacturers recommendations. Lysates of 6×10^6^ cells were used for the Ras pull-down assay.

### Flow cytometric analysis

Antibodies for flow cytometric (FACS) analysis (isotype control, Gr-1/Ly6G/C, Mac-1/CD11b) were purchased from BD Biosciences (Heidelberg, Germany) and used according to the recommendations of the manufacturer. The histograms display the geometric mean (GeoMean) of the analysed sample. The BrdU-FACS was carried out with the FITC BrdU Flow Kit (BD Pharmingen, BD Biosciences, Heidelberg, Germany) according to the manufacturers instructions.

The separation of cells positive and negative for Mac1 and c-kit, respectively was performed via the MACS-system (Miltenyi, Bergisch-Gladbach, Germany). The separation was carried out using anti-PE microbeads and MS columns in combination with Mac-1/CD11b-PE and c-kit/CD117-PE antibodies from BD Biosciences (Heidelberg, Germany) according to the instructions of the manufacturer.

### RQ–PCR and RT-PCR

RNA was isolated with PeqGold TriFast according to the manufacturers instructions (Peqlab, Erlangen, Germany). RNA (2 µg) was reverse transcribed with 200 U M-MLV-RT (Invitrogen, Karlsruhe, Germany). cDNA was amplified either by PCR using a Q-PCR kit (Immomix from Bioline, Luckenwalde, Germany) and the product detected on agarose gel, or by quantitative real-time PCR and the product detected with SYBR green using a Mx3000 (Stratagene, USA) detection system. Expression of *rps16* mRNA was used as reference. Real-time PCR was performed in triplicates and error bars indicate standard deviation. 31 primary AML cases with inversion (16), diagnosed and treated within the Germany AML-SHG 96 trial, were provided by T.I. and analyzed by RQ-PCR for *SIAT10* expression. Expression values were normalized to *β-actin* mRNA and HL60 cells as a reference.

### Mutagenesis PCR

The mutagenesis of mutated RasV12 to wild type Ras was done with Quick Change Multi Site-Directed Mutagenesis Kit from Stratagene (CA, USA) according to the protocol of the manufacturer. The sequences of primers used for the PCR reaction were as follows: forward primer, GTTGTTGTTGGCGCCGGTGGTGTGGGCAAGAGTG, reverse primer, CACTCTTGCCCACACCACCGGCGCCAACAACAAC.

### Statistical analysis

Statistical analysis was performed with two-tailed Student's t test with Welch's correction.

## Supporting Information

Figure S1 RAS does not influence the expression of MLL-ENL-ER. (A) Total RNA was isolated from control and Ras cells and after cDNA synthesis PCR with primers specific for MLL-ENL-ER was performed. Samples were analysed on an agarose gel. RT, reverse transcriptase. rps16: control gene
(0.23 MB TIF)

Figure S2 Ras cells show enhanced sensitivity to cytarabine in clonogenic assays. (A) Quantification of the colony assays shown in Figure 2E. Triplicate samples of cells incubated for 24 hours with the indicated concentrations of cytarabine were plated in methylcellulose; colonies were stained after 4 days. The plot shows the relative efficiency of colony formation of Ras versus control cells. To generate this plot, the average number of colonies formed by control cells at each concentration of cytarabine was arbitrarily set to one. (B) Reprensentative colony assays of two independently derived clones of MLL-ENL cells and their corresponding Ras cells treated for 24 hours with the indicated concentrations of cytarabine.
(1.53 MB TIF)

Figure S3 Control- and Ras cells consist of different populations. (A) Sorting of Mac1-positive vs Mac1-negative cells. The table shows the number of cells after Mac1 separation. The bulk population of control and Ras cells consist mainly of undifferentiated, Mac1-negative cells. (B) Sorting of c-kit-positive vs c-kit-negative cells. The table shows the number of cells after c-kit MACS separation. (C) 3.000 MACS separated control and Ras cells were plated in methylcellulose and colonies were stained after four days. The graph shows the number of colonies observed in three independent colony formation assays of MACS sorted cells.
(0.36 MB TIF)

Figure S4 Overexpression of wild type RAS (wt) leads to marginal activation of the checkpoint after cytarabine treatment. (A) MLL-ENL-ER cells were infected with retroviruses expressing wild type HA-RAS. Two different pools of infected cells (wt1 and wt2) expressing different levels of wild type HA-RAS were treated with 50 µM cytarabine for one hour and the immunoblots probed with the indicated antibodies. (B) RQ-PCR analysis documenting the relative mRNA expression levels of either wild type HA-RAS (wt) or oncogenic (Ras) HA-RAS.
(0.62 MB TIF)

Figure S5 Dependence of Chk1-phosphorylation and of colony formation of control and Ras cells on the concentration of cytarabine. (A) Quantification of the immunoblot shown in Figure 4A. (B) Quantification of the colony assays shown in Figure 4B. This plot shows the relative efficiency of colony formation of Ras versus control cells. To generate this plot, the average number of colonies formed by control cells at each concentration of cytarabine was arbitrarily set to one.
(0.58 MB TIF)

Figure S6 Enhanced differentiation of Ras cells in response to cytarabine. (A) Quantification of the morphological analysis shown in Figure 5A. Meta, Metamyelocyte; PMN, polymorphonuclear; Mono, Monocyte; Macro, Macrophage. (B) Control and Ras cells untreated or treated with 350 nM cytarabine for 24 hours were stained with PE-labeled antibodies against Mac1 and Gr1, respectively, and subjected to FACS analysis.
(0.92 MB TIF)

Figure S7 Caffeine abrogates the phosphorylation of Chk1. (A) The panel shows an immunoblot of Ras cells treated with 100 µM cytarabine for one hour in the presence of the indicated concentrations of caffeine; the blot was probed with antibodies against phospho-Chk1 (pSer345). (B) Immunoblots of Ras cells treated with 100 µM cytarabine for eight hours in the presence of the indicated concentrations of caffeine; the blot was probed with antibodies against p53 and p21.
(0.40 MB TIF)

Figure S8 Phosphorylation of Chk1 is independent of differentiation. The panels show immunoblots of lysates from control and Ras cells, which were cultured 12 days in the absence of 4-OHT. The indicated cell types were probed with antibodies against phospho-Chk1 (pSer345) and Cdk2. Cells were treated either with 100 µM cytarabine for the indicated times or with UV-B for 3.5 minutes and subsequently incubated for the indicated times. Exp.  =  exposure.
(0.29 MB TIF)

Figure S9 Expression of Cdk4R24C does not abrogate myeloid differentiation. (A) Cdk4R24C does not abrogate phosphorylation of Chk1. Immunoblot documenting the expression of Cdk4R24C, phospho-Chk1 (Ser345) and Ras. Ras and Ras/Cdk4RC cells were treated with 100 µM cytarabine for one hour. Cell lysates were probed for Cdk4R24C-Flag with antibodies against Flag, phospho-Chk1 (Ser345), Ras and Cdk2. (B) Phosphorylation of Rb is elevated in Ras/Cdk4R24C cells. Ras and Ras/Cdk4R24C cells were incubated with 100 µM cytarabine for the indicated times. The panels show immunoblots of cell lysates that were probed with antibodies against phospho-Rb (pSer807 and pSer811), Rb, p16Ink4a and Flag (Cdk4R24C). Cdk2 served as loading control. The asterisk denotes a non-specific band. (C) Ras cells differentiate despite expression of Cdk4R24C. Ras and Ras/Cdk4R24C cells were treated with 350 nM cytarabine for 24 hours, washed and cultured for additional two days in the absence of cytarabine. Cells were subjected to FACS analysis using α-Gr1-PE antibodies. (D) Quantification of (C). (E) Cytarabine induces the expression of ly6g mRNA in Ras/Cdk4R24C. Ras and Ras/Cdk4R24C cells were treated with 350 nM cytarabine for 24 hours and expression of ly6g mRNA was analyzed by RQ-PCR. (F) Cdk4R24C does not enhance the clonogenic potential of Ras cells. The assay was performed as described in Figure 2.
(0.81 MB TIF)

Figure S10 Expression of mdm2 is downregulated in Ras/p53DD cells. Ras and Ras/p53DD cells were treated with 350 nM cytarabine for 24 hours and expression of mdm2 mRNA was analyzed by RQ-PCR.
(0.20 MB TIF)

Figure S11 p53 is required for RAS- and cytarabine-induced differentiation. Ras and Ras/p53DD cells were treated with 350 nM cytarabine for 24 hours washed and cultured for additional two days in the absence of cytarabine. Cells were subjected to FACS analysis using α-Gr1-PE and α-Mac1-FITC antibodies, respectively.
(0.22 MB TIF)

Figure S12 Inhibition of p53 by pifithrin-α enhances the colony formation potential of Ras cells upon cytarabine treatment.(A) Cells were treated for 24 hours with 100 nM cytarabine and 20 µM pifithrin-α as indicated. Samples of 3.000 cells each were plated in methylcellulose. Colonies were stained after four days. Expression of the inactive L344P mutant of p53DD (p53LP) does not restore the clonogenic potential of Ras cells upon transient exposure to cytarabine. (B) Quantification of (A). The graph shows the number of colonies relative to the number of colonies observed in the absence of cytarabine. The graph shows the average of three independent experiments.
(1.23 MB TIF)

## References

[1] Schlenk RF, Dohner K, Krauter J, Frohling S, Corbacioglu A (2008). Mutations and treatment outcome in cytogenetically normal acute myeloid leukemia.. N Engl J Med.

[2] Tallman MS, Gilliland DG, Rowe JM (2005). Drug therapy for acute myeloid leukemia.. Blood.

[3] Frohling S, Scholl C, Gilliland DG, Levine RL (2005). Genetics of myeloid malignancies: pathogenetic and clinical implications.. J Clin Oncol.

[4] Mayer R, Davis R, Schiffer C, Berg D, Powell B (1994). Intensive postremission chemotherapy in adults with acute myeloid leukemia: Cancer and Leukemia Group B. New England Journal of Medicine.

[5] Estey E, Dohner H (2006). Acute myeloid leukaemia.. Lancet.

[6] Bos JL (1989). ras oncogenes in human cancer: A review.. Cancer Research.

[7] Larsen J, Beug H, Hayman MJ (1992). The v-ski oncogene cooperates with the v-sea oncogene in erythroid transformation by blocking erythroid differentiation.. Oncogene.

[8] MacKenzie KL, Dolnikov A, Millington M, Shounan Y, Symonds G (1999). Mutant N-ras induces myeloproliferative disorders and apoptosis in bone marrow repopulated mice.. Blood.

[9] Braun BS, Tuveson DA, Kong N, Le DT, Kogan SC (2004). Somatic activation of oncogenic Kras in hematopoietic cells initiates a rapidly fatal myeloproliferative disorder.. Proc Natl Acad Sci U S A.

[10] Chan IT, Kutok JL, Williams IR, Cohen S, Kelly L (2004). Conditional expression of oncogenic K-ras from its endogenous promoter induces a myeloproliferative disease.. J Clin Invest.

[11] Parikh C, Subrahmanyam R, Ren R (2006). Oncogenic NRAS rapidly and efficiently induces CMML- and AML-like diseases in mice.. Blood.

[12] Braun BS, Archard JA, Van Ziffle JA, Tuveson DA, Jacks TE (2006). Somatic activation of a conditional KrasG12D allele causes ineffective erythropoiesis in vivo.. Blood.

[13] Chan IT, Kutok JL, Williams IR, Cohen S, Moore S (2006). Oncogenic K-ras cooperates with PML-RAR alpha to induce an acute promyelocytic leukemia-like disease.. Blood.

[14] Hibi S, Loehler J, Friel J, Stocking C, Ostertag W (1993). Induction of monocytic differentiation and tumorigenicity by v-Ha-ras in differentiation-arrested hematopoietic cells.. Blood.

[15] Hawley RG, Fong AZ, Ngan BY, Hawley TS (1995). Hematopoietic transforming potential of activated ras in chimeric mice.. Oncogene.

[16] Darley RL, Burnett AK (1999). Mutant RAS inhibits neutrophil but not macrophage differentiation and allows continued growth of neutrophil precursors.. Exp Hematol.

[17] Shen SW, Dolnikov A, Passioura T, Millington M, Wotherspoon S (2004). Mutant N-ras preferentially drives human CD34+ hematopoietic progenitor cells into myeloid differentiation and proliferation both in vitro and in the NOD/SCID mouse.. Exp Hematol.

[18] Shen S, Passioura T, Symonds G, Dolnikov A (2007). N-ras oncogene-induced gene expression in human hematopoietic progenitor cells: upregulation of p16INK4a and p21CIP1/WAF1 correlates with myeloid differentiation.. Exp Hematol.

[19] Serrano M, Lin AW, McCurrach ME, Beach D, Lowe SW (1997). Oncogenic ras provokes premature cell senescence associated with accumulation of p53 and p16INK4a.. Cell.

[20] Groth A, Weber JD, Willumsen BM, Sherr CJ, Roussel MF (2000). Oncogenic Ras induces p19ARF and growth arrest in mouse embryo fibroblasts lacking p21Cip1 and p27Kip1 without activating cyclin D-dependent kinases.. J Biol Chem.

[21] Michaloglou C, Vredeveld LC, Soengas MS, Denoyelle C, Kuilman T (2005). BRAFE600-associated senescence-like cell cycle arrest of human naevi.. Nature.

[22] Braig M, Lee S, Loddenkemper C, Rudolph C, Peters AH (2005). Oncogene-induced senescence as an initial barrier in lymphoma development.. Nature.

[23] Collado M, Gil J, Efeyan A, Guerra C, Schuhmacher AJ (2005). Tumour biology: senescence in premalignant tumours.. Nature.

[24] Di Micco R, Fumagalli M, Cicalese A, Piccinin S, Gasparini P (2006). Oncogene-induced senescence is a DNA damage response triggered by DNA hyper-replication.. Nature.

[25] Fikaris AJ, Lewis AE, Abulaiti A, Tsygankova OM, Meinkoth JL (2006). Ras triggers ataxia-telangiectasia-mutated and Rad-3-related activation and apoptosis through sustained mitogenic signaling.. J Biol Chem.

[26] Neubauer A, Maharry K, Mrozek K, Thiede C, Marcucci G (2008). Patients with acute myeloid leukemia and RAS mutations benefit most from postremission high-dose cytarabine: A Cancer and Leukemia Group B study.. J Clin Oncol.

[27] Zeisig BB, Garcia-Cuellar MP, Winkler TH, Slany RK (2003). The oncoprotein MLL-ENL disturbs hematopoietic lineage determination and transforms a biphenotypic lymphoid/myeloid cell.. Oncogene.

[28] Zeisig BB, Milne T, Garcia-Cuellar MP, Schreiner S, Martin ME (2004). Hoxa9 and Meis1 are key targets for MLL-ENL-mediated cellular immortalization.. Mol Cell Biol.

[29] Collado M, Serrano M (2006). The power and the promise of oncogene-induced senescence markers.. Nat Rev Cancer.

[30] Kamijo T, Zindy F, Roussel MF, Quelle DE, Downing JR (1997). Tumor suppression at the mouse INK4a locus mediated by the alternative reading frame product p19ARF.. Cell.

[31] Bartkova J, Horejsi Z, Koed K, Kramer A, Tort F (2005). DNA damage response as a candidate anti-cancer barrier in early human tumorigenesis.. Nature.

[32] Shen WPV, Alfrich TH, Venta-Perez G, Franza BR, Furth ME (1987). Expression of normal and mutant ras proteins in human acute leukemia.. Oncogene.

[33] Nagler A, Kletter Y, Ricklis I, Gazit E, Tatarsky I (1986). Effect of cytosine arabinoside on differentiation of normal human bone marrow cells.. Exp Hematol.

[34] Sarkaria JN, Busby EC, Tibbetts RS, Roos P, Taya Y (1999). Inhibition of ATM and ATR kinase activities by the radiosensitizing agent, caffeine.. Cancer Res.

[35] Ivanova NB, Dimos JT, Schaniel C, Hackney JA, Moore KA (2002). A stem cell molecular signature.. Science.

[36] Subramanian A, Tamayo P, Mootha VK, Mukherjee S, Ebert BL (2005). Gene set enrichment analysis: a knowledge-based approach for interpreting genome-wide expression profiles.. Proc Natl Acad Sci U S A.

[37] Wolfel T, Hauer M, Schneider J, Serrano M, Wolfel C (1995). A p16INK4a-insensitive CDK4 mutant targeted by cytolytic T lymphocytes in a human melanoma.. Science.

[38] Gottlieb E, Haffner R, von Ruden T, Wagner EF, Oren M (1994). Down-regulation of wild-type p53 activity interferes with apoptosis of IL-3-dependent hematopoietic cells following IL-3 withdrawal.. Embo J.

[39] Komarov PG, Komarova EA, Kondratov RV, Christov-Tselkov K, Coon JS (1999). A chemical inhibitor of p53 that protects mice from the side effects of cancer therapy.. Science.

[40] Vassilev LT, Vu BT, Graves B, Carvajal D, Podlaski F (2004). In vivo activation of the p53 pathway by small-molecule antagonists of MDM2.. Science.

[41] Kojima K, Konopleva M, Samudio IJ, Shikami M, Cabreira-Hansen M (2005). MDM2 antagonists induce p53-dependent apoptosis in AML: implications for leukemia therapy.. Blood.

[42] Kojima K, Konopleva M, McQueen T, O'Brien S, Plunkett W (2006). Mdm2 inhibitor Nutlin-3a induces p53-mediated apoptosis by transcription-dependent and transcription-independent mechanisms and may overcome Atm-mediated resistance to fludarabine in chronic lymphocytic leukemia.. Blood.

[43] Gu L, Zhu N, Findley HW, Zhou M (2008). MDM2 antagonist nutlin-3 is a potent inducer of apoptosis in pediatric acute lymphoblastic leukemia cells with wild-type p53 and overexpression of MDM2.. Leukemia.

[44] Secchiero P, Zerbinati C, Melloni E, Milani D, Campioni D (2007). The MDM-2 antagonist nutlin-3 promotes the maturation of acute myeloid leukemic blasts.. Neoplasia.

[45] Radich JP, Kopecky KJ, Appelbaum F, Willman CL, Collins SJ (1992). N-ras mutations in acute myelogeneous leukemia: A review of the current literature and an update ot the Southwest Oncology Group experience.. Leukemia and Lymphoma.

[46] Paquette RL, Landaw EM, Pierre RV, Kahan J, Luebbert M (1993). N-ras mutations are associated with poor prognosis and increased risk of leukemia in myelodysplastic syndromes.. Blood.

[47] Coghlan D, Morley AA, Matthews JP, Bishop JF (1994). The incidence and prognostic significance of mutations in codon 13 of the N-ras gene in acute myeloid leukemia.. Leukemia.

[48] Neubauer A, Dodge R, George SL, Davey FR, Silver RT (1994). Prognostic importance of mutations in the ras protooncogenes in de novo acute myeloid leukemia.. Blood.

[49] Illmer T, Thiede C, Fredersdorf A, Stadler S, Neubauer A (2005). Activation of the RAS pathway is predictive for a chemosensitive phenotype of acute myelogenous leukemia blasts.. Clin Cancer Res.

[50] Bacher U, Haferlach T, Schoch C, Kern W, Schnittger S (2006). Implications of NRAS mutations in AML: a study of 2502 patients.. Blood.

[51] Cozzio A, Passegue E, Ayton PM, Karsunky H, Cleary ML (2003). Similar MLL-associated leukemias arising from self-renewing stem cells and short-lived myeloid progenitors.. Genes Dev.

[52] Barabe F, Kennedy JA, Hope KJ, Dick JE (2007). Modeling the initiation and progression of human acute leukemia in mice.. Science.

[53] Capizzi RL (1996). Curative chemotherapy for acute myeloid leukemia: the development of high-dose ara-C from the laboratory to bedside.. Invest New Drugs.

[54] Abulaiti A, Fikaris AJ, Tsygankova OM, Meinkoth JL (2006). Ras induces chromosome instability and abrogation of the DNA damage response.. Cancer Res.

[55] Huang CL, Deng ML, Guo RJ, Wu MT, Liu FZ (1988). A study on the induction of differentiation of human leukemic cells by harringtonine combined with cytarabine.. Leukemia.

[56] Wiederschain D, Kawai H, Shilatifard A, Yuan ZM (2005). Multiple mixed lineage leukemia (MLL) fusion proteins suppress p53-mediated response to DNA damage.. J Biol Chem.

[57] Potenza N, Vecchione C, Notte A, De Rienzo A, Rosica A (2005). Replacement of K-Ras with H-Ras supports normal embryonic development despite inducing cardiovascular pathology in adult mice.. EMBO Rep.

[58] Schreiner S, Birke M, Garcia-Cuellar MP, Zilles O, Greil J (2001). MLL-ENL causes a reversible and myc-dependent block of myelomonocytic cell differentiation.. Cancer Res.

[59] Dimri GP, Lee X, Basile G, Acosta M, Scott G (1995). A biomarker that identifies senescent human cells in culture and in aging skin in vivo.. Proc Natl Acad Sci U S A.

