# Supplementary figures and images for: Oncogenic RAS Enables DNA Damage- and p53-Dependent Differentiation of Acute Myeloid Leukemia Cells in Response to Chemotherapy
Source: PLoS One. 2009 Nov 5;4(11):e7768. doi: 10.1371/journal.pone.0007768 (PMC2767509; doi:10.1371/journal.pone.0007768)

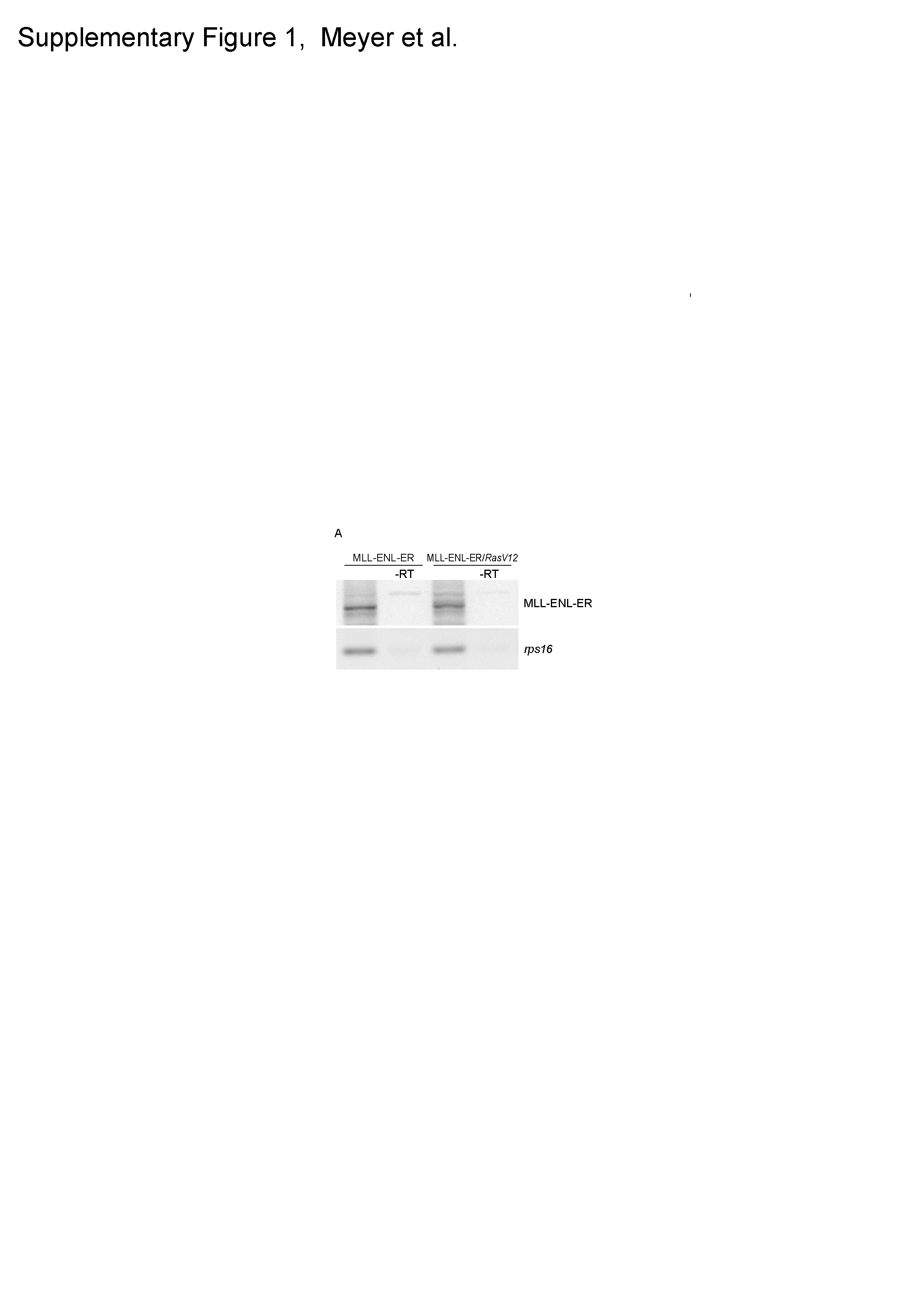

Supplement: Figure S1 — RAS does not influence the expression of MLL-ENL-ER. (A) Total RNA was isolated from control and Ras cells and after cDNA synthesis PCR with primers specific for MLL-ENL-ER was performed. Samples were analysed on an agarose gel. RT, reverse transcriptase. rps16: control gene (0.23 MB TIF) [file pone.0007768.s001.tif]

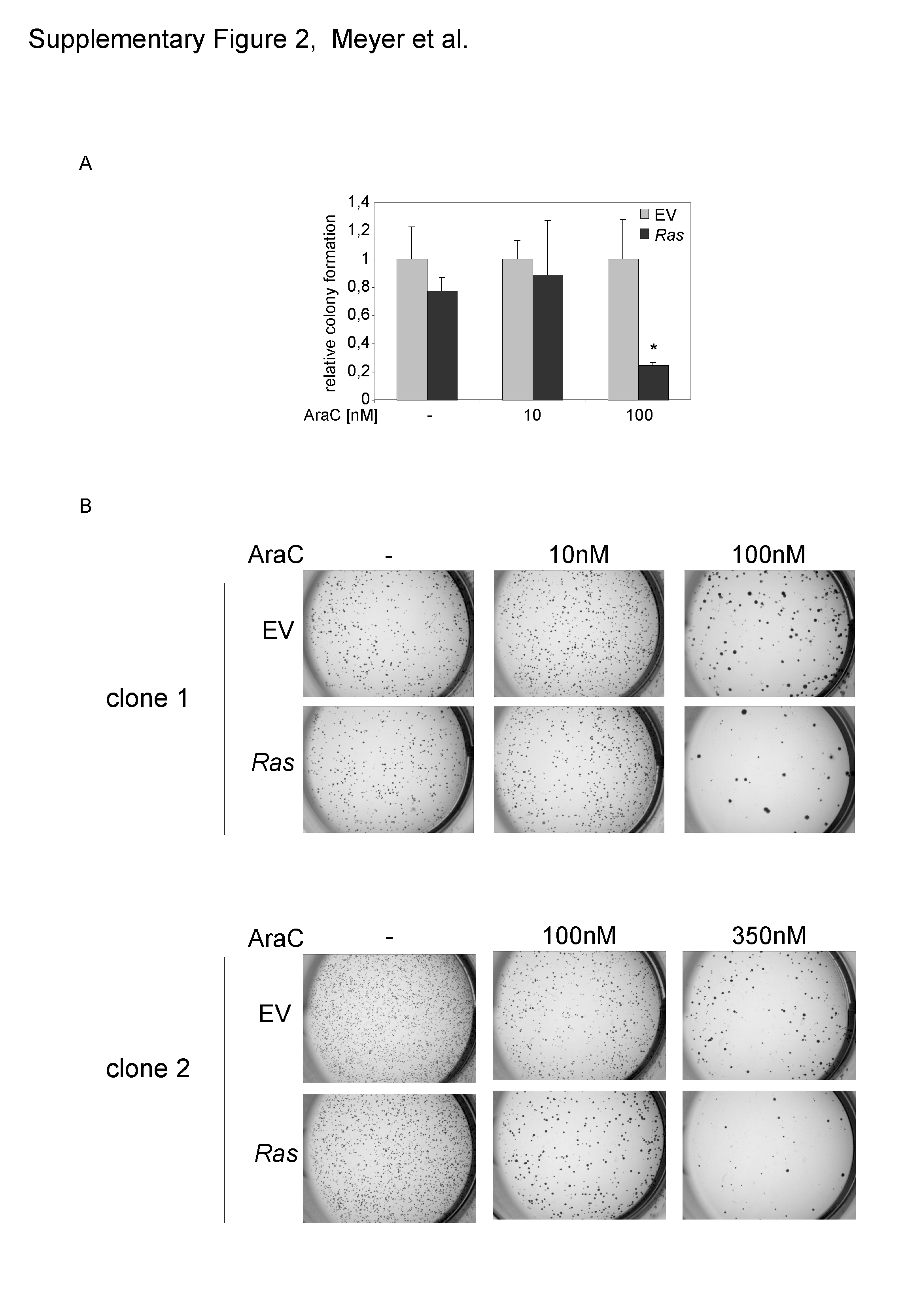

Supplement: Figure S2 — Ras cells show enhanced sensitivity to cytarabine in clonogenic assays. (A) Quantification of the colony assays shown in Figure 2E. Triplicate samples of cells incubated for 24 hours with the indicated concentrations of cytarabine were plated in methylcellulose; colonies were stained after 4 days. The plot shows the relative efficiency of colony formation of Ras versus control cells. To generate this plot, the average number of colonies formed by control cells at each concentration of cytarabine was arbitrarily set to one. (B) Reprensentative colony assays of two independently derived clones of MLL-ENL cells and their corresponding Ras cells treated for 24 hours with the indicated concentrations of cytarabine. (1.53 MB TIF) [file pone.0007768.s002.tif]

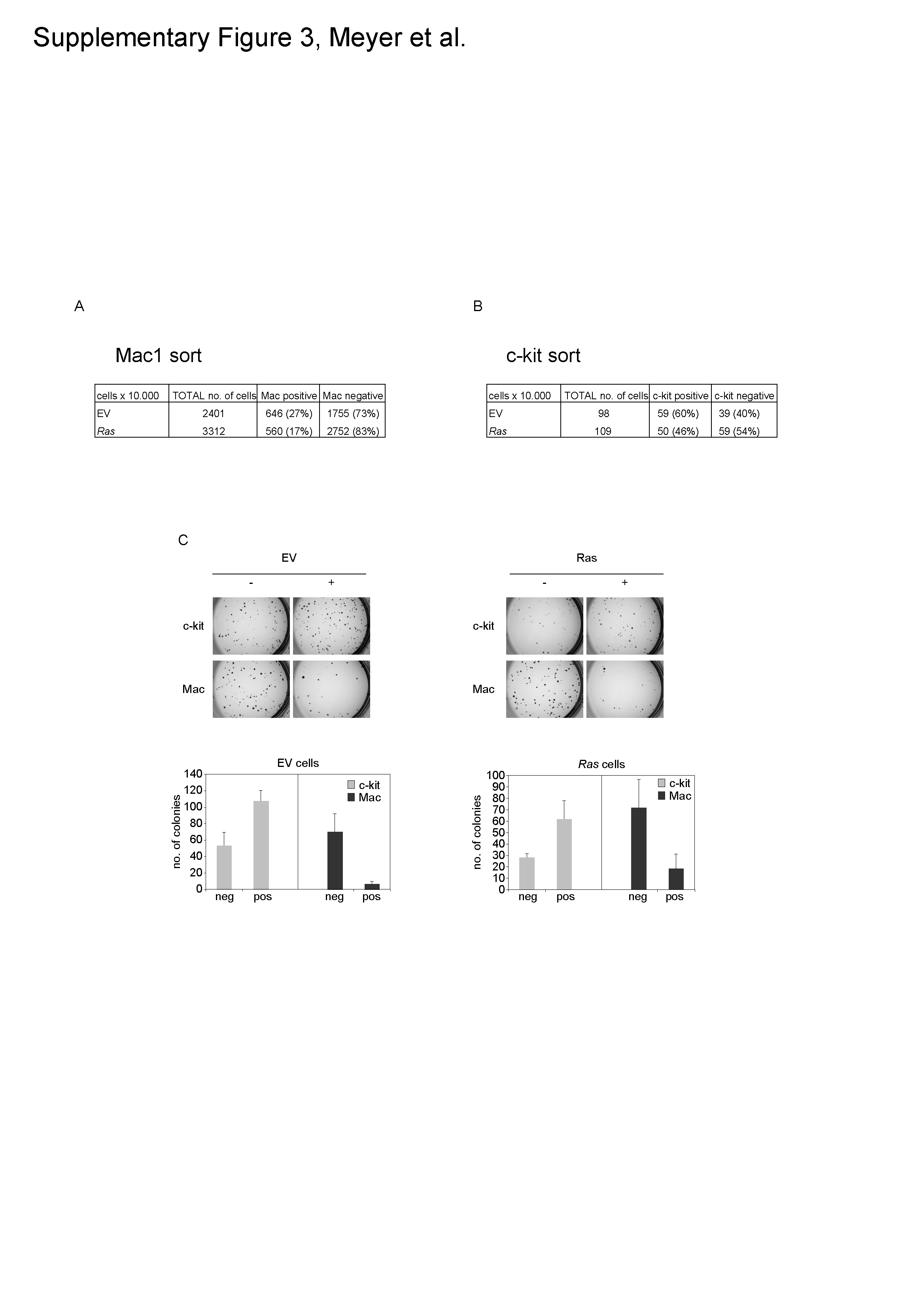

Supplement: Figure S3 — Control- and Ras cells consist of different populations. (A) Sorting of Mac1-positive vs Mac1-negative cells. The table shows the number of cells after Mac1 separation. The bulk population of control and Ras cells consist mainly of undifferentiated, Mac1-negative cells. (B) Sorting of c-kit-positive vs c-kit-negative cells. The table shows the number of cells after c-kit MACS separation. (C) 3.000 MACS separated control and Ras cells were plated in methylcellulose and colonies were stained after four days. The graph shows the number of colonies observed in three independent colony formation assays of MACS sorted cells. (0.36 MB TIF) [file pone.0007768.s003.tif]

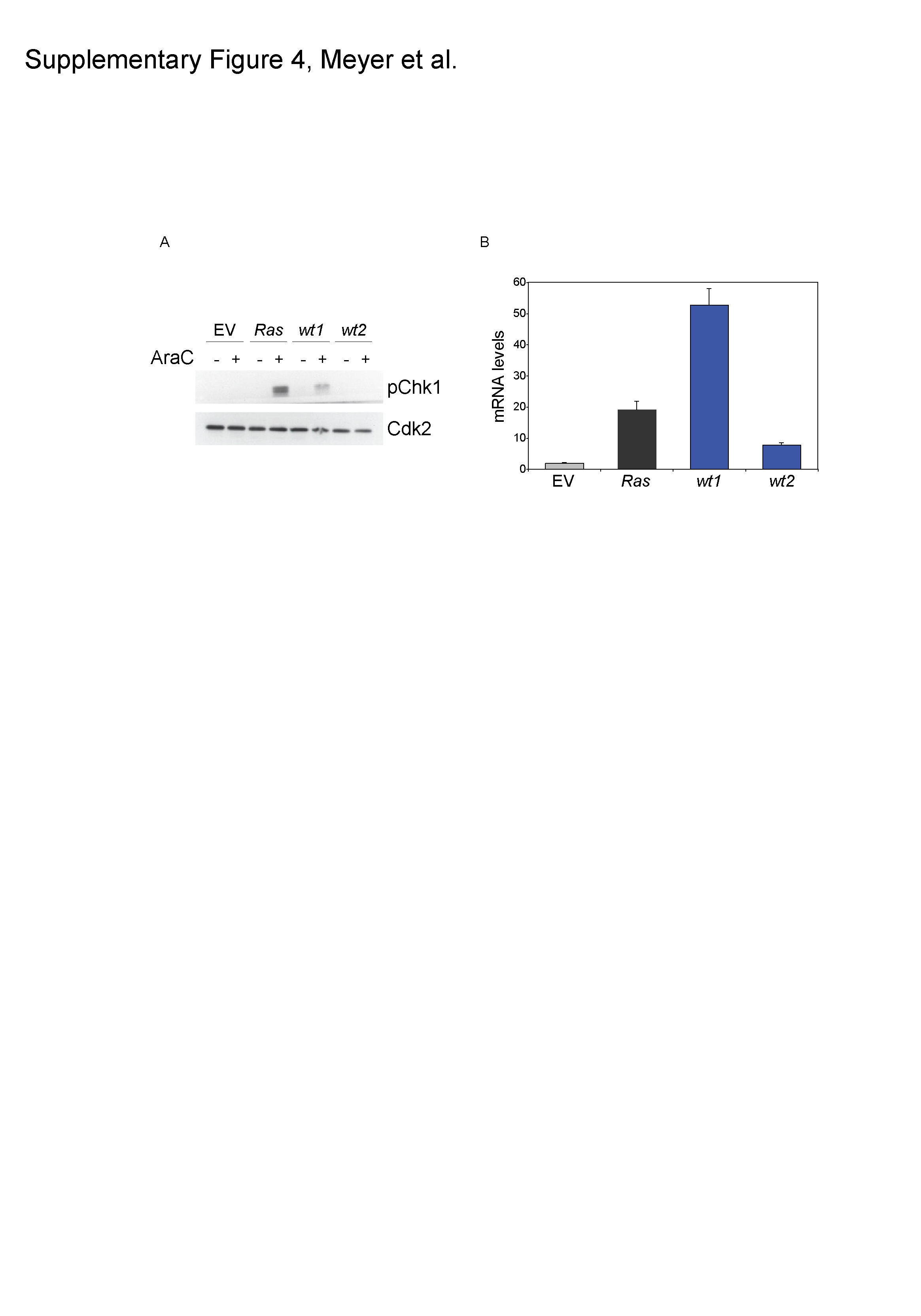

Supplement: Figure S4 — Overexpression of wild type RAS (wt) leads to marginal activation of the checkpoint after cytarabine treatment. (A) MLL-ENL-ER cells were infected with retroviruses expressing wild type HA-RAS. Two different pools of infected cells (wt1 and wt2) expressing different levels of wild type HA-RAS were treated with 50 µM cytarabine for one hour and the immunoblots probed with the indicated antibodies. (B) RQ-PCR analysis documenting the relative mRNA expression levels of either wild type HA-RAS (wt) or oncogenic (Ras) HA-RAS. (0.62 MB TIF) [file pone.0007768.s004.tif]

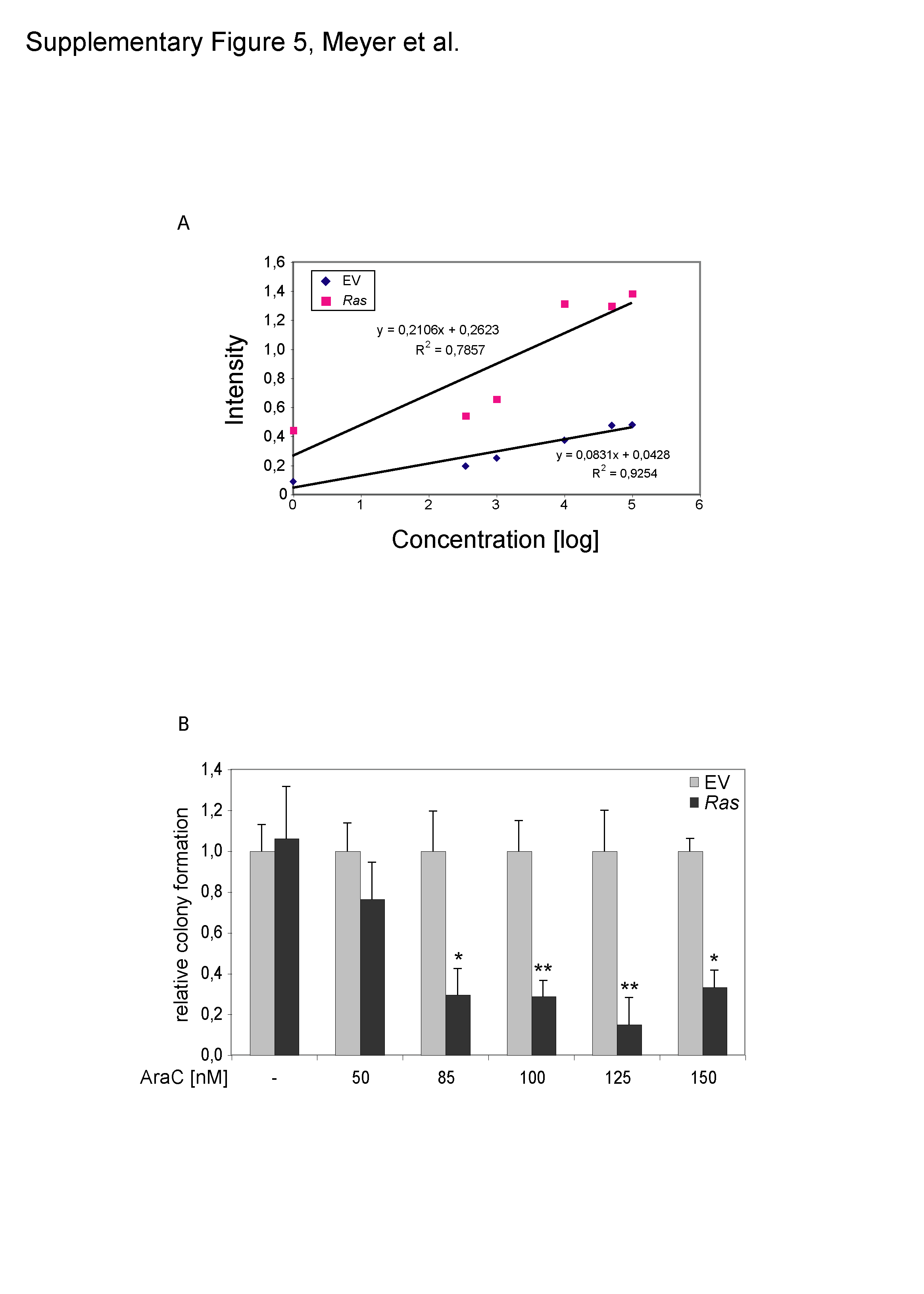

Supplement: Figure S5 — Dependence of Chk1-phosphorylation and of colony formation of control and Ras cells on the concentration of cytarabine. (A) Quantification of the immunoblot shown in Figure 4A. (B) Quantification of the colony assays shown in Figure 4B. This plot shows the relative efficiency of colony formation of Ras versus control cells. To generate this plot, the average number of colonies formed by control cells at each concentration of cytarabine was arbitrarily set to one. (0.58 MB TIF) [file pone.0007768.s005.tif]

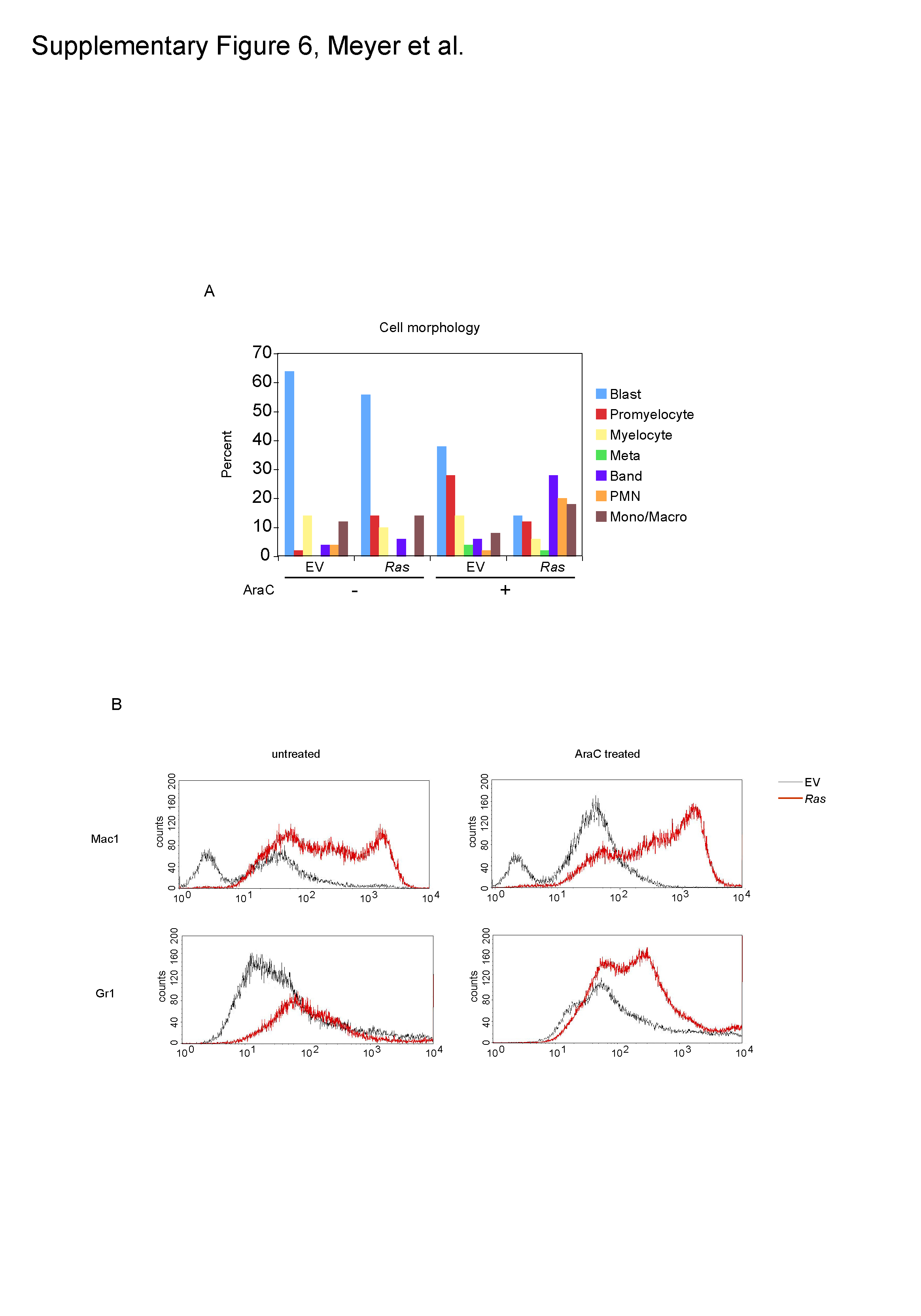

Supplement: Figure S6 — Enhanced differentiation of Ras cells in response to cytarabine. (A) Quantification of the morphological analysis shown in Figure 5A. Meta, Metamyelocyte; PMN, polymorphonuclear; Mono, Monocyte; Macro, Macrophage. (B) Control and Ras cells untreated or treated with 350 nM cytarabine for 24 hours were stained with PE-labeled antibodies against Mac1 and Gr1, respectively, and subjected to FACS analysis. (0.92 MB TIF) [file pone.0007768.s006.tif]

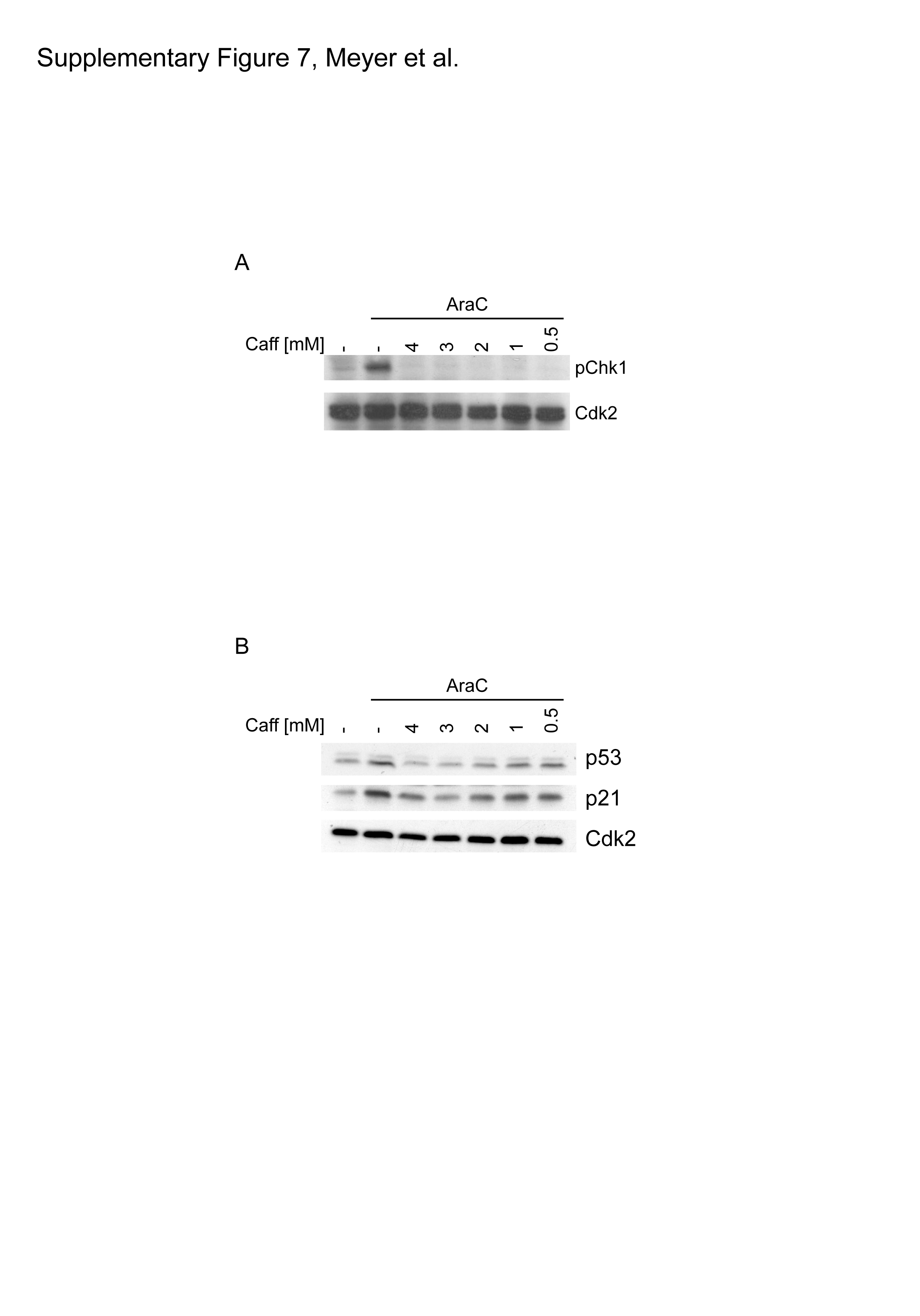

Supplement: Figure S7 — Caffeine abrogates the phosphorylation of Chk1. (A) The panel shows an immunoblot of Ras cells treated with 100 µM cytarabine for one hour in the presence of the indicated concentrations of caffeine; the blot was probed with antibodies against phospho-Chk1 (pSer345). (B) Immunoblots of Ras cells treated with 100 µM cytarabine for eight hours in the presence of the indicated concentrations of caffeine; the blot was probed with antibodies against p53 and p21. (0.40 MB TIF) [file pone.0007768.s007.tif]

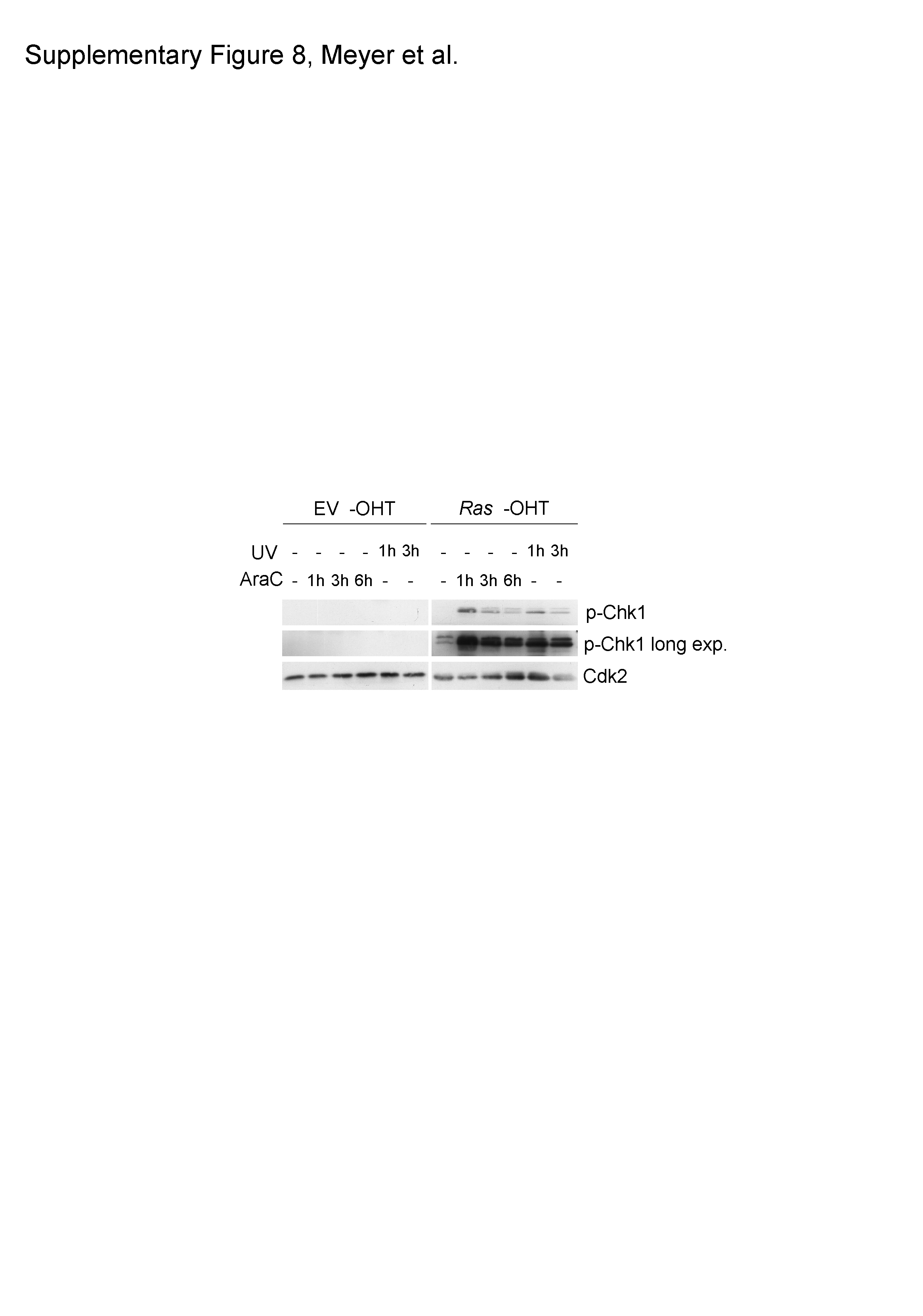

Supplement: Figure S8 — Phosphorylation of Chk1 is independent of differentiation. The panels show immunoblots of lysates from control and Ras cells, which were cultured 12 days in the absence of 4-OHT. The indicated cell types were probed with antibodies against phospho-Chk1 (pSer345) and Cdk2. Cells were treated either with 100 µM cytarabine for the indicated times or with UV-B for 3.5 minutes and subsequently incubated for the indicated times. Exp. = exposure. (0.29 MB TIF) [file pone.0007768.s008.tif]

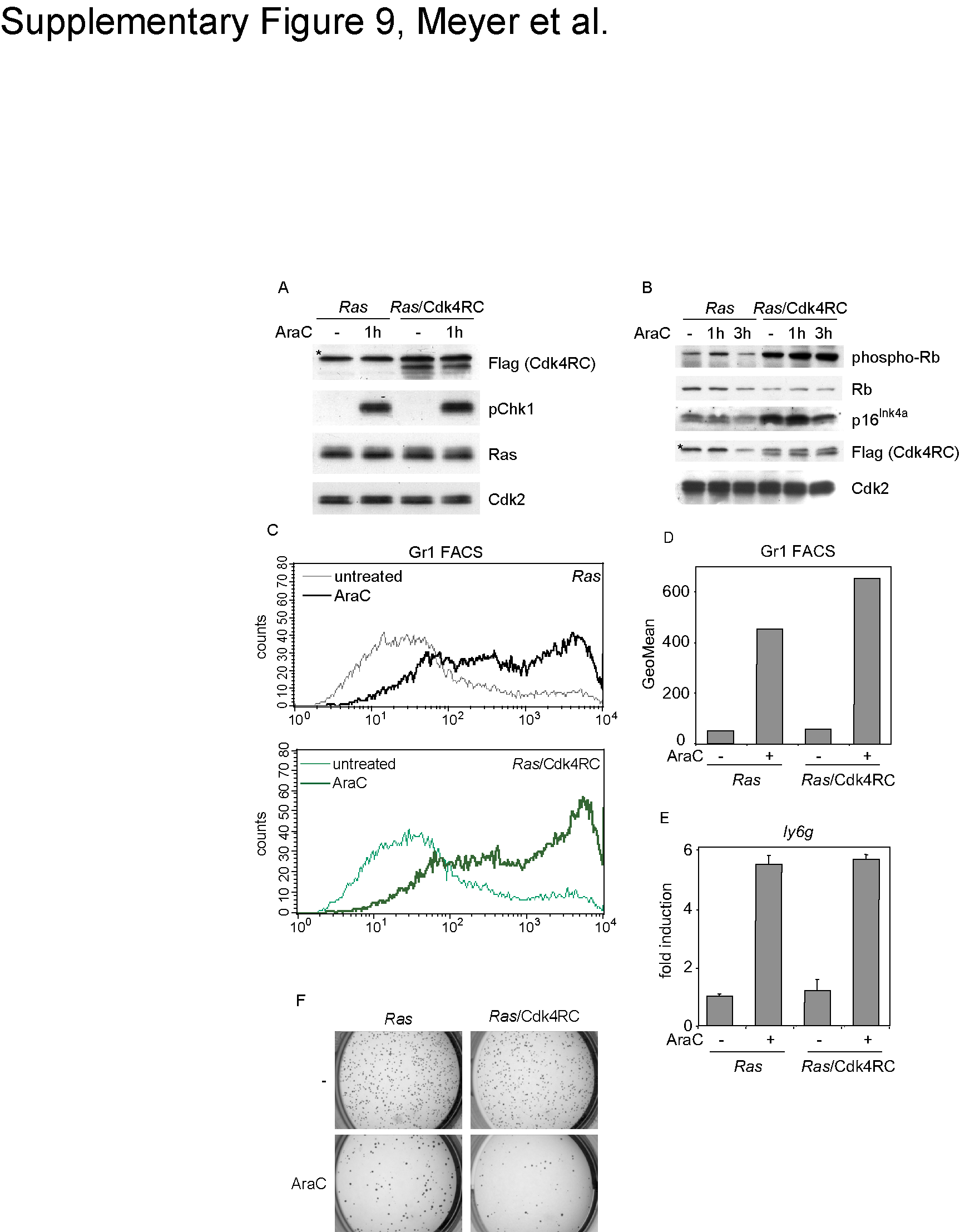

Supplement: Figure S9 — Expression of Cdk4R24C does not abrogate myeloid differentiation. (A) Cdk4R24C does not abrogate phosphorylation of Chk1. Immunoblot documenting the expression of Cdk4R24C, phospho-Chk1 (Ser345) and Ras. Ras and Ras/Cdk4RC cells were treated with 100 µM cytarabine for one hour. Cell lysates were probed for Cdk4R24C-Flag with antibodies against Flag, phospho-Chk1 (Ser345), Ras and Cdk2. (B) Phosphorylation of Rb is elevated in Ras/Cdk4R24C cells. Ras and Ras/Cdk4R24C cells were incubated with 100 µM cytarabine for the indicated times. The panels show immunoblots of cell lysates that were probed with antibodies against phospho-Rb (pSer807 and pSer811), Rb, p16Ink4a and Flag (Cdk4R24C). Cdk2 served as loading control. The asterisk denotes a non-specific band. (C) Ras cells differentiate despite expression of Cdk4R24C. Ras and Ras/Cdk4R24C cells were treated with 350 nM cytarabine for 24 hours, washed and cultured for additional two days in the absence of cytarabine. Cells were subjected to FACS analysis using α-Gr1-PE antibodies. (D) Quantification of (C). (E) Cytarabine induces the expression of ly6g mRNA in Ras/Cdk4R24C. Ras and Ras/Cdk4R24C cells were treated with 350 nM cytarabine for 24 hours and expression of ly6g mRNA was analyzed by RQ-PCR. (F) Cdk4R24C does not enhance the clonogenic potential of Ras cells. The assay was performed as described in Figure 2. (0.81 MB TIF) [file pone.0007768.s009.tif]

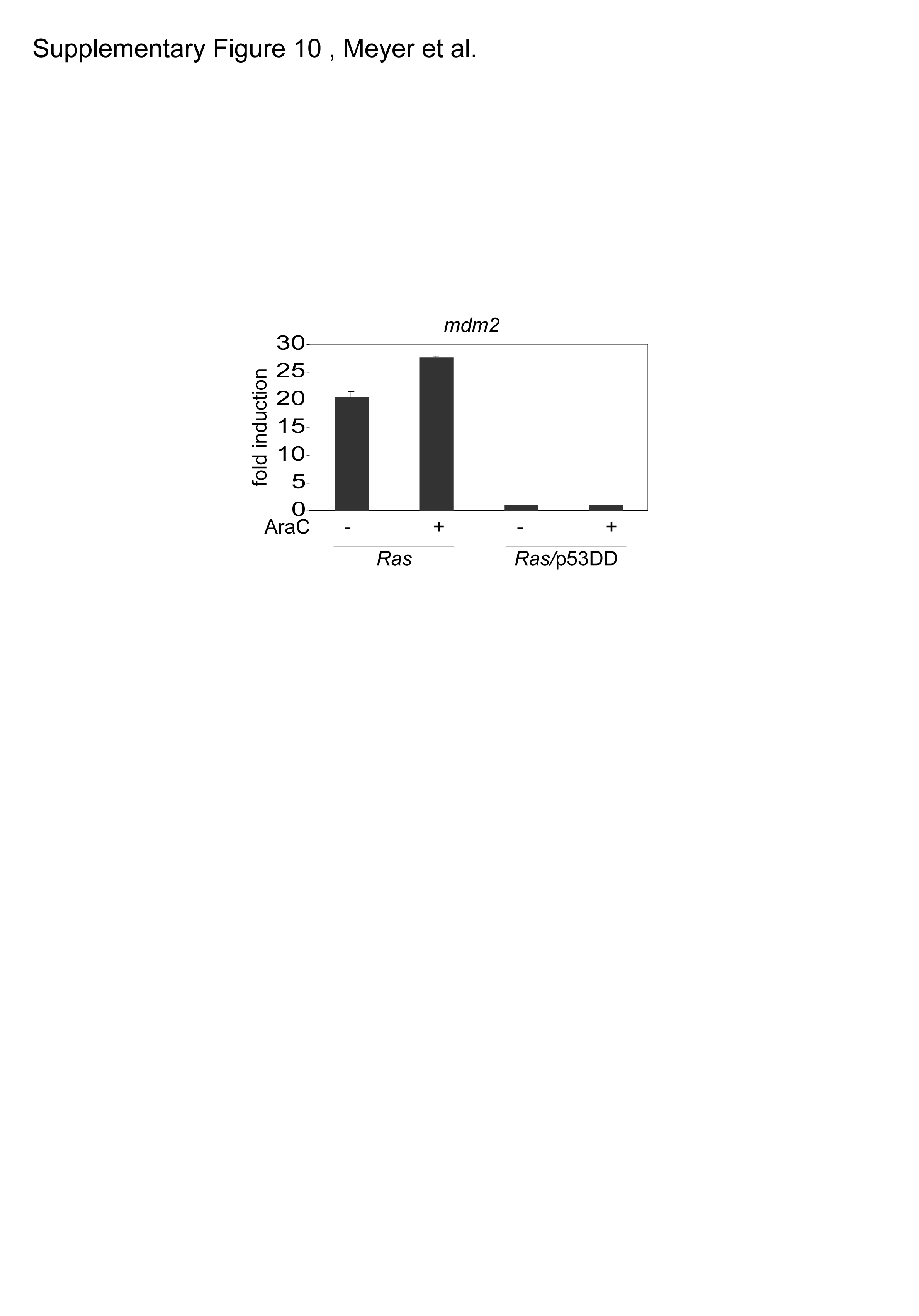

Supplement: Figure S10 — Expression of mdm2 is downregulated in Ras/p53DD cells. Ras and Ras/p53DD cells were treated with 350 nM cytarabine for 24 hours and expression of mdm2 mRNA was analyzed by RQ-PCR. (0.20 MB TIF) [file pone.0007768.s010.tif]

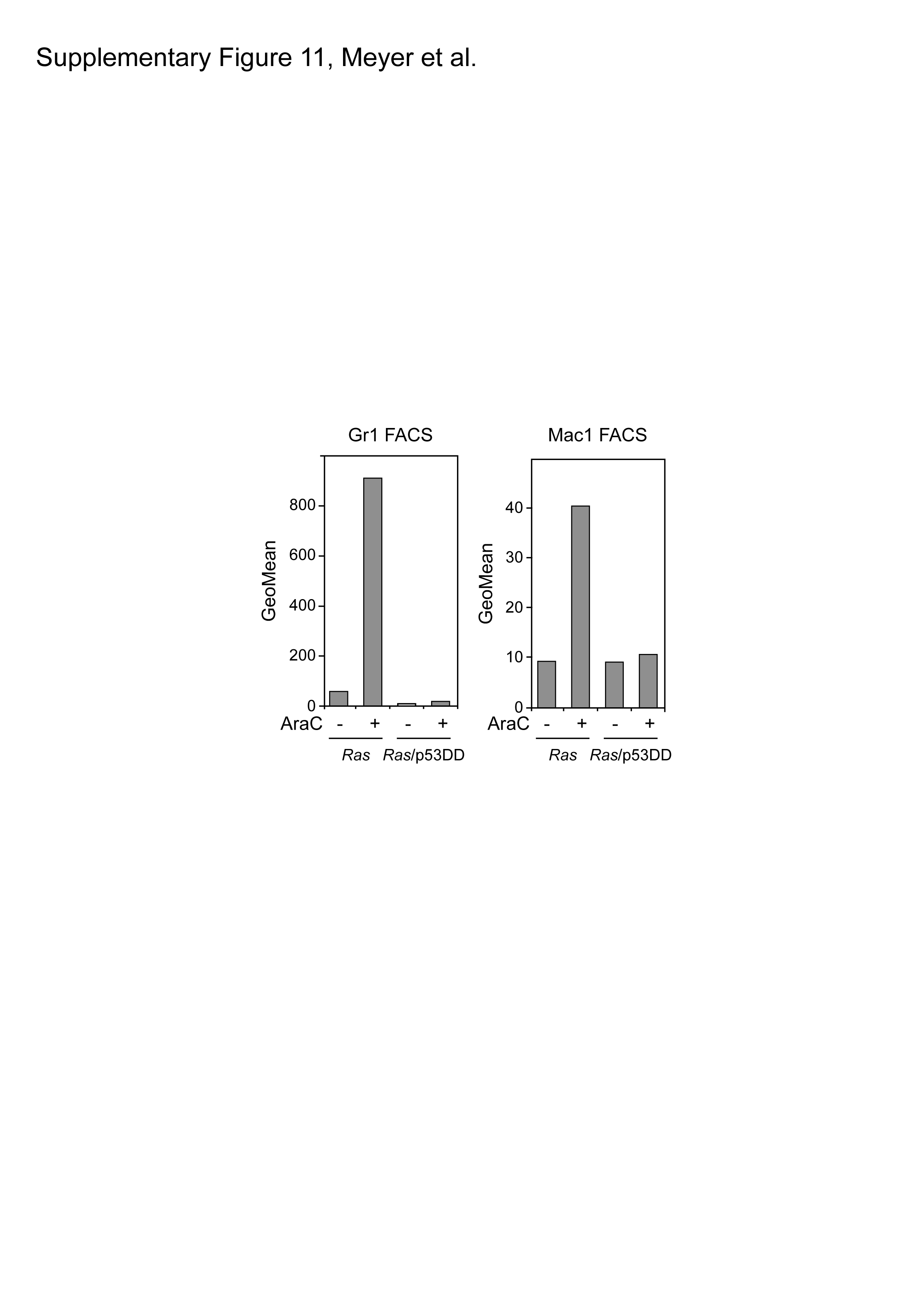

Supplement: Figure S11 — p53 is required for RAS- and cytarabine-induced differentiation. Ras and Ras/p53DD cells were treated with 350 nM cytarabine for 24 hours washed and cultured for additional two days in the absence of cytarabine. Cells were subjected to FACS analysis using α-Gr1-PE and α-Mac1-FITC antibodies, respectively. (0.22 MB TIF) [file pone.0007768.s011.tif]

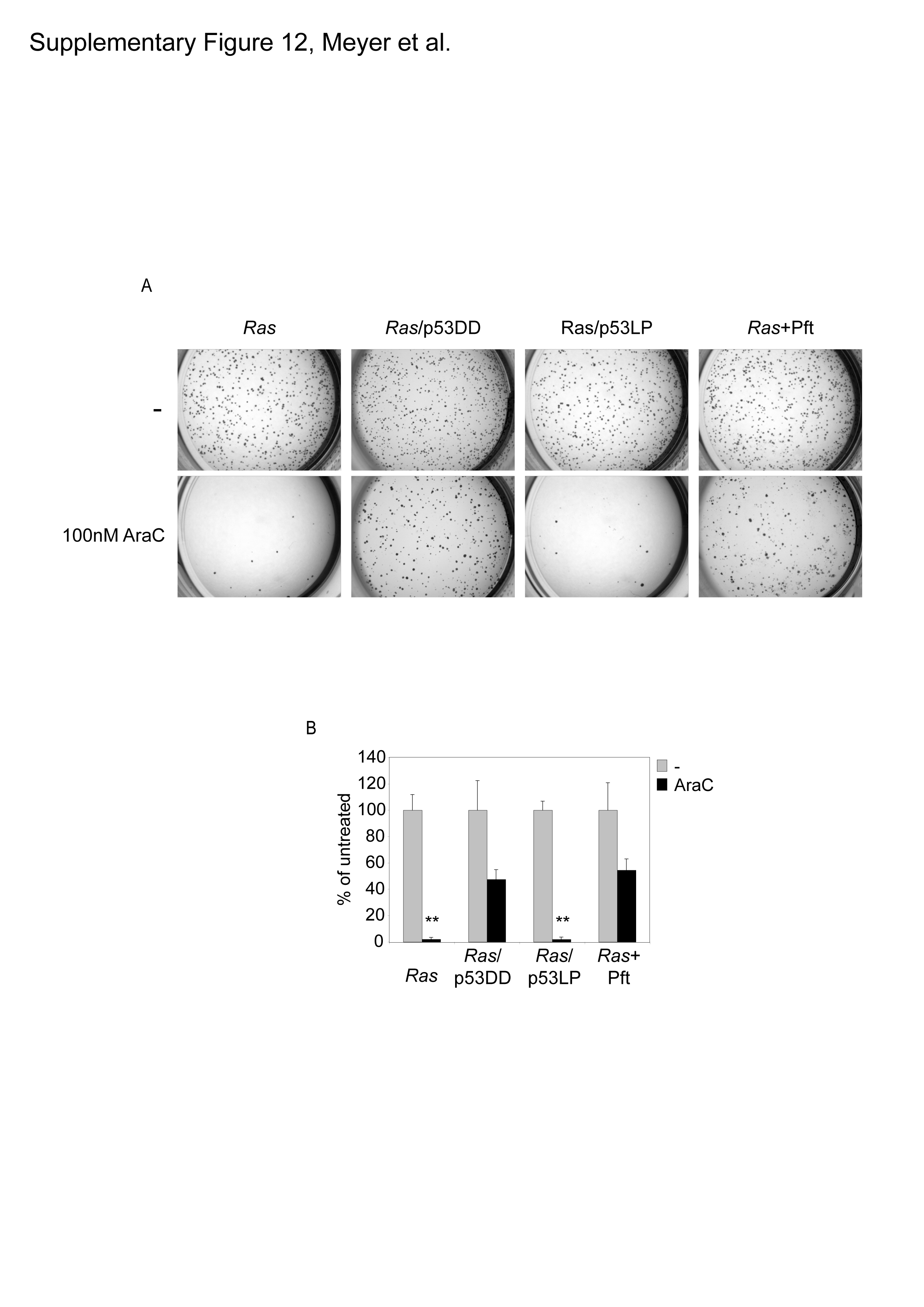

Supplement: Figure S12 — Inhibition of p53 by pifithrin-α enhances the colony formation potential of Ras cells upon cytarabine treatment.(A) Cells were treated for 24 hours with 100 nM cytarabine and 20 µM pifithrin-α as indicated. Samples of 3.000 cells each were plated in methylcellulose. Colonies were stained after four days. Expression of the inactive L344P mutant of p53DD (p53LP) does not restore the clonogenic potential of Ras cells upon transient exposure to cytarabine. (B) Quantification of (A). The graph shows the number of colonies relative to the number of colonies observed in the absence of cytarabine. The graph shows the average of three independent experiments. (1.23 MB TIF) [file pone.0007768.s012.tif]
